# A systematic analysis of protein palmitoylation in *Caenorhabditis elegans*

**DOI:** 10.1186/1471-2164-15-841

**Published:** 2014-10-02

**Authors:** Matthew J Edmonds, Alan Morgan

**Affiliations:** Department of Cellular and Molecular Physiology, Institute of Translational Medicine, University of Liverpool, Crown St., Liverpool, L69 3BX UK

**Keywords:** DHHC, Palmitoyl acyl-transferase, Palmitoyl-protein thioesterase, Neuronal ceroid lipofuscinosis

## Abstract

**Background:**

Palmitoylation is a reversible post-translational protein modification which involves the addition of palmitate to cysteine residues. Palmitoylation is catalysed by the DHHC family of palmitoyl-acyl transferases (PATs) and reversibility is conferred by palmitoyl-protein thioesterases (PPTs). Mutations in genes encoding both classes of enzymes are associated with human diseases, notably neurological disorders, underlining their importance. Despite the pivotal role of yeast studies in discovering PATs, palmitoylation has not been studied in the key animal model *Caenorhabditis elegans*.

**Results:**

Analysis of the *C. elegans* genome identified fifteen PATs, using the DHHC cysteine-rich domain, and two PPTs, by homology. The twelve uncategorised PATs were officially named using a *dhhc-x* system. Genomic data on these palmitoylation enzymes and those in yeast, *Drosophila* and humans was collated and analysed to predict properties and relationships in *C. elegans*. All available *C. elegans* strains containing a mutation in a palmitoylation enzyme were analysed and a complete library of RNA interference (RNAi) feeding plasmids against PAT or PPT genes was generated. To test for possible redundancy, double RNAi was performed against selected closely related PATs and both PPTs. Animals were screened for phenotypes including size, longevity and sensory and motor neuronal functions. Although some significant differences were observed with individual mutants or RNAi treatment, in general there was little impact on these phenotypes, suggesting that genetic buffering exists within the palmitoylation network in worms.

**Conclusions:**

This study reports the first characterisation of palmitoylation in *C. elegans* using both *in silico* and in vivo approaches, and opens up this key model organism for further detailed study of palmitoylation in future.

**Electronic supplementary material:**

The online version of this article (doi:10.1186/1471-2164-15-841) contains supplementary material, which is available to authorized users.

## Background

Palmitoylation, specifically *S*-palmitoylation, is the post-translational modification of proteins with the 16-carbon fatty acid palmitate. Unlike other lipid modifications of proteins, palmitoylation is reversible. The formation of its thioester link to cysteine residues is catalysed by palmitoyl acyl-transferases (PATs), also known as DHHCs after their conserved active site aspartic acid-histidine-histidine-cysteine motif, and the removal of palmitate is catalysed by palmitoyl-protein thioesterases (PPTs) [1–3]. Palmitoylation occurs on diverse substrates, both soluble and transmembrane, in a variety of cell types and tissues. As such, it is important in the regulation of many cellular processes, including Ras signalling and synaptic plasticity, and altered palmitoylation is associated with various human diseases, notably neurological disorders [4–6].

Although palmitoylation as a post-translational modification in eukaryotic cells had been known about for many years [7], it was not until relatively recently that the enzymes involved were identified [1, 8–10]. The realisation that the DHHCs shared their eponymous motif in a cysteine-rich domain (DHHC-CRD) allowed easy searching of whole genome data for putative DHHCs. This method has allowed the prediction of 23 such genes in humans [6, 9], 24 in mice [4], 22 in *Drosophila* [11] and eight predicted, of which seven are confirmed, in yeast [10]. The DHHC motif is also the active site of these enzymes [12] and many of the predicted DHHCs have since been validated experimentally as having palmitoyltransferase enzymatic activity [13].

The PPTs present more of a challenge to study systematically due to the lack of a common identifying motif. The mammalian cytosolic PPT acyl-protein thioesterase 1 (APT1) was originally discovered through its action in removing [^3^H]-palmitate from Gα subunits [14] and has homology to another PPT, APT2. PPT1 was discovered through its biochemical activity against palmitoylated H-Ras [15] and has been shown to be localised to lysosomes [16] or synaptic vesicles and synaptosomes [17] instead of the cytoplasm. A second lysosomal enzyme, PPT2, was found through homology to PPT1 but has not been shown to have activity against palmitoyl-proteins [3, 18].

Numerous proteome-scale studies in recent years have enabled the identification of proteins which undergo palmitoylation in different cell types and organisms [19–24]. However, there is comparatively little known about which enzyme(s) palmitoylate or depalmitoylate which substrate(s). This is partly due to the workload in characterising individual enzymes or substrates to obtain this specific information. Identification of these enzyme-substrate pairs can be better directed by examining the effects of disruption of each enzyme to show which cellular pathways they may regulate. The nematode worm *C. elegans* is a useful experimental tool for probing functions of genes in well conserved processes using simple assays. As well as mutant strains, individual gene function may be disrupted by knocking down expression using RNA interference (RNAi). This can be performed simply by feeding animals with bacteria expressing double-stranded RNA (dsRNA) against the gene of interest [25, 26].

There has been some confusion in the literature about *C. elegans* DHHCs, with figures of 15 [4], 16 [1] and 17 [27] genes being quoted. However, there has been no definitive analysis of putative DHHC enzymes or any assessment of their palmitoylating activity. The only member of the DHHC family of enzymes which has been studied in *C. elegans* is SPE-10. The *spe-10* gene was named after a spermatogenesis defect in fibrous body-membranous organelles, where the protein is localised [27, 28]. There are some stress resistance phenotypes which have been observed in *spe-10* mutants, including an increase in lifespan in some, though not all, experiments and increased resistance to UV exposure and high temperature (35°C) but not paraquat, a reactive oxygen species generator [29].

The literature on PPTs in *C. elegans* is limited to *ppt-1* [30], with no studies on its APT1 orthologue *ath-1*. PPT-1 was shown to have similar activity to the human orthologue [31] and knockouts have a number of mild phenotypes including delays in the developmental moult from the final larval stage, L4, into adults and onset of egg-laying [30]. Electron microscopy showed defects in mitochondria in energetically active tissues, including neurons [30]. Given the suitability of *C. elegans* as a model system for probing questions about both development and neuronal function [32, 33], coupled with the importance of palmitoylation in these systems [4, 30, 34], a more thorough analysis could open up new avenues of research within the field.

In this study, the *C. elegans* DHHC enzymes were first defined. *In silico* sequence analysis was performed to show the differing relationships between the DHHCs and PPTs both in reference to other members of the families in *C. elegans* and those in other organisms. A screen conducted using both mutant strains and an RNAi knockdown approach was performed to assess effects on gross phenotypes relating to basic parameters and behaviours of *C. elegans*. These data will serve as a useful platform for other researchers wishing to study palmitoylation in vivo in this simple model organism.

## Results

### Identification and naming of the *C. elegans*DHHC enzyme family

Before exploring palmitoylation in *C. elegans*, it was first necessary to identify its DHHC enzymes and gain clues as to possible phenotypes from what is already known about them and their counterparts in other organisms. A search of the specialist database WormBase (version WS207) for the Pfam tag assigned to the DHHC motif uncovered 15 genes. Four of these 15 were previously named: *dhhc-1*, *dhhc-2*, *tag-233* and *spe-10*. The remainder were annotated only with their gene code. These were renamed in line with official conventions using the *dhhc-x* nomenclature previously started. *tag-233*, a temporarily assigned gene name, was also renamed. Where possible, the number assigned to the *dhhc-x* nomenclature was based on homology to the *H. sapiens* DHHCs (Additional file 1). The *spe-10* name is based upon a published spermatogenesis defect phenotype [27–29] and so was not altered to avoid confusion within the literature. The old and new gene names and their respective codes are shown in Table 1 and the collated information available on WormBase is in Additional file 2. These changes in gene nomenclature were effective from release WS235 of WormBase.

**Table 1:** ***C. elegans*** **strain and RNAi clone availability**

Gene	Gene code	Available mutant(s) (***allele***)	RNAi clone source
*dhhc-1*	F09B12.2	**tm4272 (** ***tm4272*** **)**	Vidal library
*dhhc-2*	Y47H9C.2	**RB1044 (** ***ok990*** **)**	Vidal library
*dhhc-3*	F33D11.12		In-house
*dhhc-4*	ZK757.4		In-house
*dhhc-5*	R13F6.5		Vidal library
*dhhc-6*	M18.8		In-house
*dhhc-7*	C17D12.1		Vidal library
*dhhc-8*	Y39E4B.7		In-house
*dhhc-9*	C43H6.7	**VC2067 (** ***gk985*** **)**	Vidal library
*dhhc-10*	K02G10.1		Vidal library
*dhhc-11*	T22E7.2		Vidal library
*dhhc-12*	F59C6.2	**VC2039 (** ***gk1013*** **), VC2244 (** ***gk981*** **)**	Vidal library
*dhhc-13*	H32C10.3	**VC108 (** ***gk36*** **)**	In-house
*dhhc-14*	D2021.2	**VC771 (** ***gk330*** **), VC918 (** ***ok1032*** **)**	In-house
*spe-10*	AC3.10	**BA744 (** ***hc104*** **)**	In-house
*ath-1*	K04G2.5	**RB1484 (** ***ok1735*** **)**	Vidal library
*ppt-1*	F44C4.5	**MN1 (** ***gk139*** **), VC166 (** ***gk131*** **), VC168 (** ***gk134*** **)**, VC183 (*gk139*), VC184 (*gk140*)	Vidal library

The DHHC (*dhhc-1* to -*14* and *spe-10*) and PPT (*ath-1* and *ppt-1*) genes and their sequence codes in *C. elegans* are listed. If a *C. elegans* strain containing a mutation in the gene-of-interest only is available, it is shown here along with the allele identifier in parentheses. Strains shown in bold were used in this study. More detailed information on the mutations can be found in Additional file 5. RNAi clones were either obtained from the Vidal *C. elegans* ORF RNAi Library [39] or made in-house. *ath*, acyl-protein thioesterase; *spe*, spermatogenesis-deficient.

### Sequence analysis

**Figure 1:**
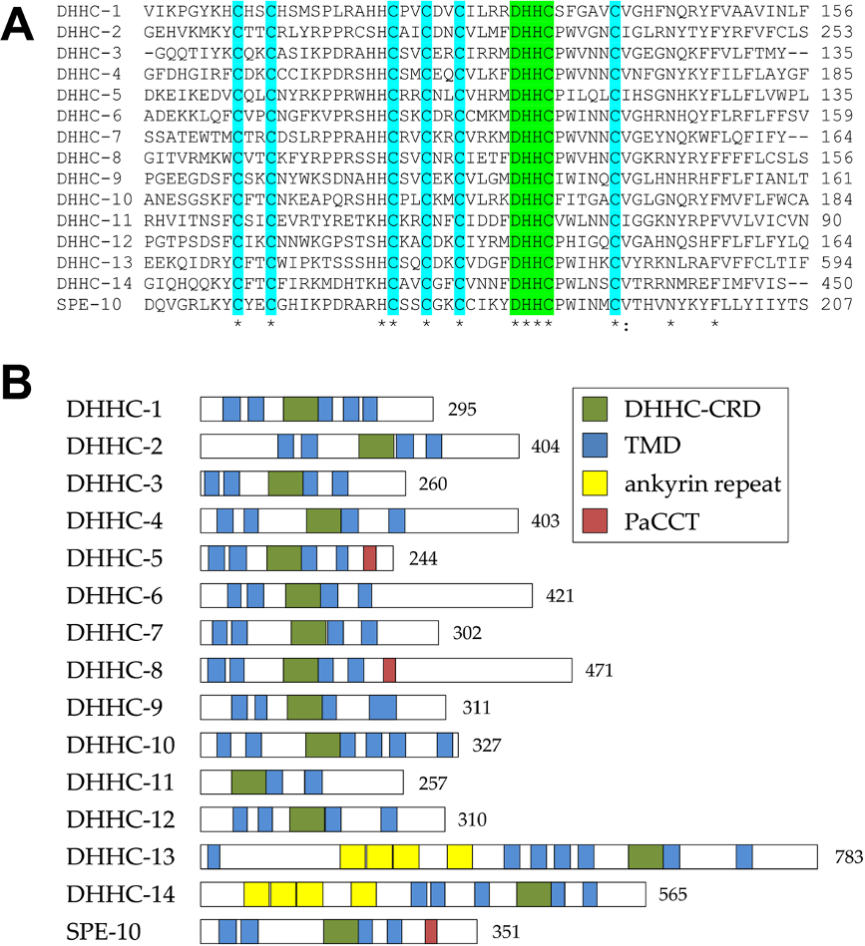
**Sequence analysis of the putative** ***C. elegans*** **DHHC enzymes. (A)** An alignment of the cysteine-rich domain (DHHC-CRD) of the putative *C. elegans* DHHC enzymes. The absolutely conserved DHHC motif is highlighted in green and conserved cysteine residues in cyan. The position of the final residue in the sequence shown is indicated on the right. **(B)** The predicted domain structure of the *C. elegans* DHHCs is represented in schematics drawn to scale. Features indicated are the DHHC-CRD (green), transmembrane domains (blue), ankyrin repeats (yellow) and palmitoyltransferase conserved C-terminal (PaCCT) (red). The sequence length in amino acids is indicated on the right.

**Figure 2:**
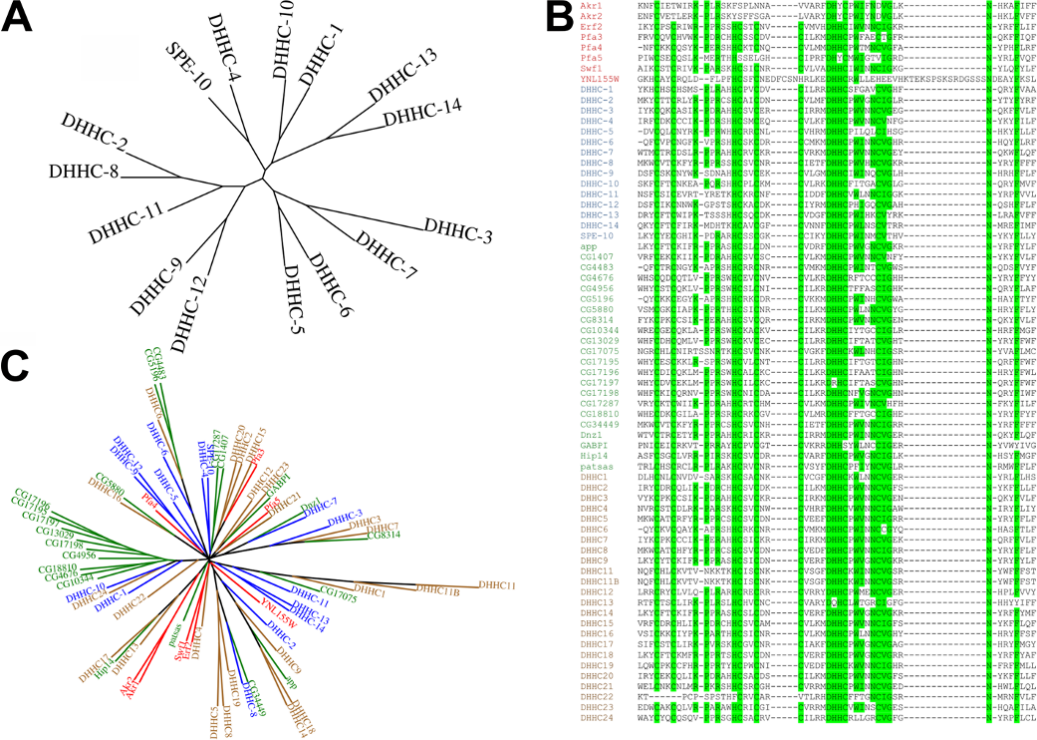
**Phylogenetic analysis of DHHC enzymes from yeast, worms, fruit flies and humans. (A)** The 15 DHHCs from *C. elegans* were subjected to phylogenetic analysis and the resulting trees were rendered using Interactive Tree of Life [71]. **(B)** An alignment of the DHHC-motif cysteine-rich domain (DHHC-CRD) of the known DHHC enzymes from *S. cerevisiae* (red lettering), *C. elegans* (blue), *D. melanogaster* (green) and *H. sapiens* (brown) is shown. Specific residues in the consensus sequence Cx_2_Cx_3_(R/K)PxRx_2_HCx_2_Cx_2_Cx_4_DHHCxW(V/I)xNC(I/V)Gx_2_Nx_3_F [1] are highlighted in light green. **(C)** The conserved DHHC-CRD from these organisms **(C)** was also subjected to phylogenetic analysis. Species and colours are as in panel **(B)**.

A sequence alignment of the primary sequence of the 15 *C. elegans* DHHCs confirms that all of them contain the DHHC motif required for enzymatic activity within a cysteine-rich domain (Figure 1A). The positions of these cysteines relative to the DHHC motif are absolutely conserved and correspond to those originally observed in this domain [8]. To assess whether the *C. elegans* DHHCs conform to the common structure found in this family, a domain structure diagram was constructed using domains predicted from the primary sequence (Figure 1B). In general, DHHCs have been found to have four transmembrane domains (TMDs), though some have more, and the DHHC-CRD is normally located in a cytoplasmic loop between the second and third TMDs [1, 4, 6, 35]. The majority of the *C. elegans* DHHCs fit this description, although there are some exceptions. DHHC-11 has no TMDs preceding the DHHC-CRD, while DHHC-13 and -14 have five and three TMDs respectively preceding the DHHC-CRD. DHHC-13 and -14 are the only *C. elegans* DHHCs which contain multiple ankyrin repeats in the N-terminal section of the sequence, and these enzymes show closest homology to the mammalian DHHCs containing this domain (Additional file 1). Three enzymes, DHHC-5 and -8 and SPE-10, also contain a palmitoyltransferase conserved C-terminal (PaCCT) motif. This motif is only present in a subset of DHHC enzymes across organisms, but has been shown to be required for the activity of the *S. cerevisiae* DHHCs Swf1 and Pfa3 [36].The protein sequences were also subjected to a phylogenetic analysis. The enzymes mostly form pairs which are closely related on the resulting phylogenetic tree (Figure 2A) and some of the features correlate well with the domain structures (Figure 1B). For example the ankyrin repeat-containing proteins DHHC-13 and -14 are one such pair. The exception is DHHC-11, which can probably be accounted for by its lack of two N-terminal TMDs compared with the rest of the family.

Given the lack of experimental information on the vast majority of the *C. elegans* DHHCs, they were compared with the DHHC family in other model organisms and humans. The sequences of the conserved DHHC-CRD region of DHHC enzymes from *S. cerevisiae*, *D. melanogaster* and *H. sapiens* were combined with the *C. elegans* sequences for a large scale analysis. Whilst the consensus sequence for DHHCs is quite precisely defined, it has been noted that there are variations within it which seem to be tolerated [1]. With this in mind, the DHHC sequences were aligned with the focus on the region containing the full consensus sequence (Figure 2B). There does indeed seem to be variation in how important individual residues in the consensus sequence are. The only residues which are absolutely conserved are the aspartate of the DHHC motif and the asparagine which is the penultimate residue in the sequence. Although absolutely conserved among the *C. elegans* DHHCs, many of the cysteine residues which form the CRD are missing from some enzymes from other species. Even the DHHC motif, required for enzymatic activity, is not absolutely conserved. The *S. cerevisiae* DHHCs Akr1, Akr2 and Pfa5 have a tyrosine instead of a histidine residue in the third position, although these enzymes retain their palmitoyltransferase activity [1]. The histidine residue in the second position is changed to an arginine in *D. melanogaster* CG17197 and glutamine in *H. sapiens* DHHC13 (also called Huntingtin-interacting protein 14-like (HIP14L)). Again, while the former has not been studied, HIP14L is known to have palmitoyltransferase activity [37]. The cysteine in the DHHC motif is changed to a serine residue in the *D. melanogaster* DHHC β1,4-N-acetylgalactosaminyl-transferase B pilot (GABPI). Given that this cysteine residue is required to form the palmitoyl-enzyme intermediate [12] and its mutation to serine is a common way to inactivate DHHCs, it is likely that GABPI has no palmitoyltransferase activity in vivo. In a phylogenetic tree using the DHHC-CRD of DHHCs from these same organisms (Figure 2C), the *C. elegans* DHHCs retain the pairs seen on the phylogenetic tree of their full-length sequences, and their altered positions in relation to each other are likely to reflect differing areas of similarity around the DHHC-CRD compared with the N- and C-termini. The *C. elegans* DHHCs also appear mostly to be related, albeit distantly in some cases, to proteins in other organisms, allowing predictions to be made based on knowledge from other organisms.

In addition to the DHHC-CRD, there are two other regions of relatively high sequence conservation which have been observed in DHHCs. There is an aspartate-proline-glycine (DPG) motif commonly next to TMD2 and a threonine-threonine-x-glutamate (TTxE) motif commonly next to TMD4 [1]. The DPG motif can be found in *C. elegans* DHHC-2, -3, -5 and -7, and the TTxE motif in *C. elegans* DHHC-3, -4, -6 and -10 and SPE-10. The functional significance of these motifs is not yet known.

### The *C. elegans*palmitoyl-protein thioesterases

Unlike the DHHCs, there are no unifying motifs in the PPTs which allow easy genomic searches. This limits the process of finding PPTs in unexplored genomes to searching for orthologues of known PPTs. The two known sub-families of PPTs, the PPTs and the APTs, are well represented across diverse organisms, although not many have both types of PPT. There is generally a reasonable degree of sequence conservation within orthologues of each individual protein, as can be seen in sequence alignments (Additional file 3). However, there is no one conserved region in common between the two sequences. In *C. elegans* there are two PPTs, PPT-1 and ATH-1, which are orthologues of *H. sapiens* PPT1 and APT1 respectively.

The sequences of the PPTs in *S. cerevisiae*, *C. elegans*, *D. melanogaster* and *H. sapiens* were subjected to phylogenetic analysis and a phylogenetic tree was constructed, showing a clear divide between the PPT and APT sub-families (Additional file 4). The APT sub-family seems to be derived from the *S. cerevisiae* thioesterase enzyme Apt1, whereas the PPT subfamily is only present in the more complex organisms. Apt1 was shown by a systematic GFP-tagging study [38] to be localised to the cytoplasm and nucleus in yeast. This is consistent with the known localisation of *H. sapiens* APT1 in the cytoplasm [14], compared with PPT1 and -2, which are lysosomal [3, 16]. It is therefore likely that the *C. elegans* enzymes will follow a similar pattern, with PPT-1 lysosomal and ATH-1 cytoplasmic.

### Derivation of mutant strains and feeding RNAi clones

By collating the *C. elegans* DHHC and PPT enzymes and examining their sequences properties *in silico*, several potential relationships between these enzymes and with those in other organisms were revealed. Having assessed their predicted properties, the next step was to assess whether disruption of their expression by mutation or RNAi knockdown causes readily observable phenotypes.

WormBase and the National Bioresource Project for the Experimental Animal “Nematode *C. elegans*” website (http://www.shigen.nig.ac.jp/c.elegans/index.jsp) were used to find existing strains containing a unique mutation only in that gene (Table 1, Additional file 5). An alternative to using mutant strains is to knockdown expression of proteins simply through feeding RNAi. For this purpose, we used the Vidal *C. elegans* open reading frame (ORF) RNAi Library of bacterial RNAi feeding strains [39], which contains ten of the 15 DHHCs and both PPTs. The genes not present in the Vidal library were cloned into the empty vector to give a complete set of RNAi clones for the DHHC and PPT families (Table 1). Despite the effectiveness of RNAi in many tissues, *C. elegans* neurons in particular can be refractory to its effects [40]. A strain deficient for *rrf-3* (*pk1426* allele, designated NL2099), which suppresses amplification of secondary siRNAs, shows a general increase in sensitivity to RNAi, including in neurons [41] and so was used as the feeding RNAi background strain in this study.

### Morphology

All strains appeared to have normal morphology, behaviour and fertility during standard culture conditions. To quantify their morphology they were analysed using WormTracker software, which automatically locates an animal on a video feed of a plate, assigns a skeleton of points along the body and uses this to give precise morphological measurements [42, 43]. Wild-type N2 animals had an average length of 1222 μm and width of 78 μm (Additional file 6). Of the mutant strains tested, only *ppt-1* (*gk134*) had a significant difference in length from N2, reduced to 915 μm, but this was not observed in the other *ppt-1* alleles. Mutants for *dhhc-1* and -*12* (*gk1013*) showed a small but significant reduction in width to around 70 μm, and *dhhc-9*, -*13*, and *spe-10* showed an increase to 85–90 μm.

When *rrf-3* RNAi-hypersensitive mutants were fed negative control bacteria, which had no insert in the pG-L4440 expression vector (L4440), the animals were on average 1445 μm long and 86 μm wide (Additional file 7). There is no reported figure for the length of *rrf-3* mutants in the literature, although no obvious morphological differences were reported when this strain was first published [41] and on the plates they appear identical to N2 animals by eye. When *rrf-3* animals were treated with RNAi against a selection of genes, no significant differences in length or width were observed.

### Behavioural assays

**Figure 3:**
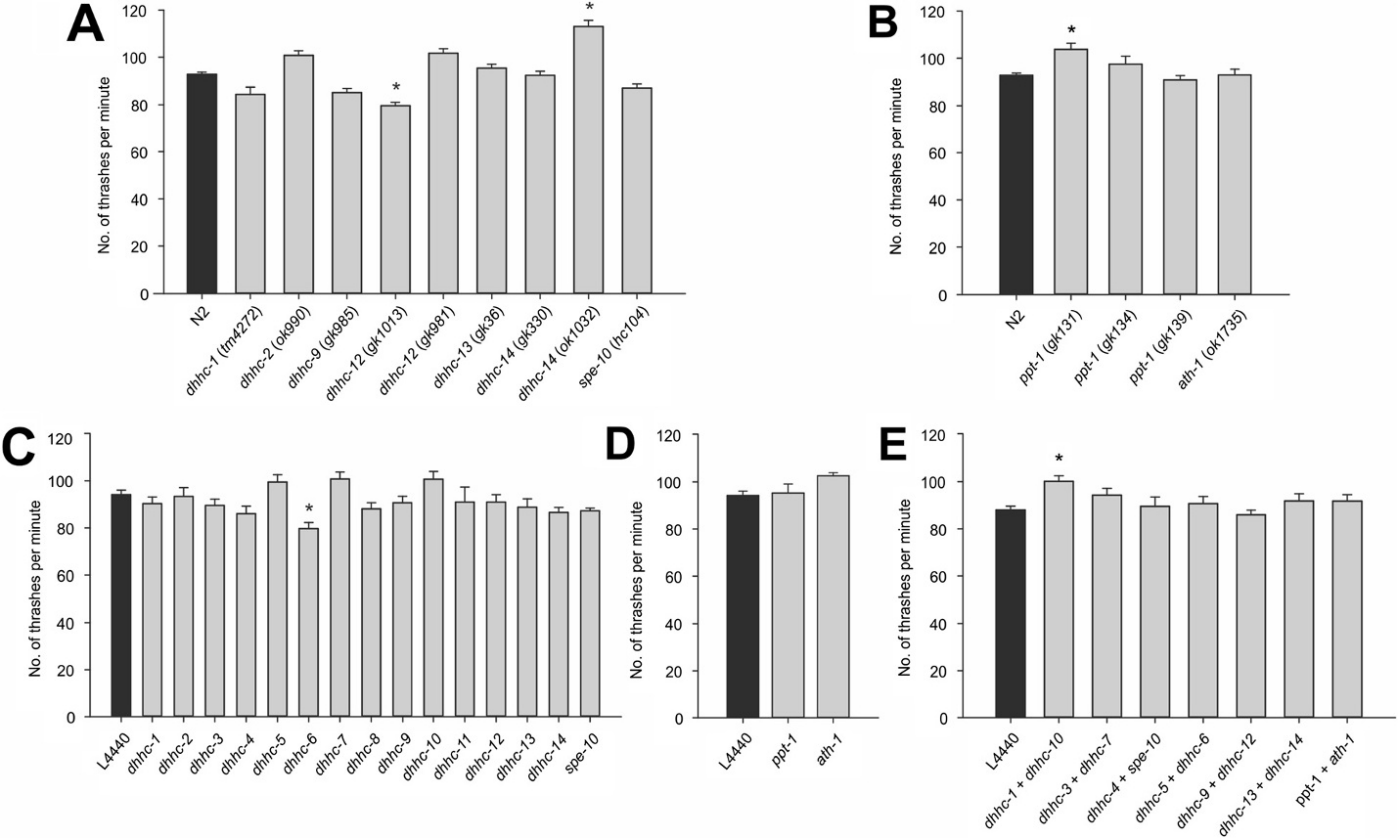
**Locomotive behaviour in solution is mostly unaffected by disruption of palmitoylation enzymes.** Thrashing assays were performed on animals deficient for DHHC enzymes, either mutant strains **(A)** or feeding RNAi-treated hypersensitive *rrf-3* mutants **(C)** and compared with wild-type Bristol N2 or empty L4440 vector control respectively. Similarly, thrashing assays were performed on mutant strains **(B)** and feeding RNAi-treated *rrf-3* mutants **(D)** for the PPT enzymes. **(E)** Closely related pairs of enzymes were knocked down by feeding RNAi and subjected to thrashing assays. *n* = 30–288 animals, from 3–14 independent experiments for mutants; and 10–49 animals, from 1–5 independent experiments for RNAi. *p < 0.05 by one-way ANOVA.

Locomotion is a measure of overall neuronal and muscular function and can be assessed in solution [44] or on solid media [45]. All mutant strains were subjected to thrashing assays in solution (Figure 3A, B). One of the *dhhc-12* mutant strains (*gk1013*) showed a significant decrease in locomotion, and *dhhc-14* (*ok1032*) and *ppt-1* (*gk131*) showed a significant increase. However, in each case the strain(s) containing the other allele available for the gene showed a non-significant difference in the opposite direction. The most likely explanation for this discrepancy is the presence of secondary mutations as a result of the mutagenesis process; it seems unlikely that similar alleles should produce such disparate effects. Thrashing assays were also carried out on RNAi-treated *rrf-3* mutants covering all the DHHC (Figure 3C) and PPT (Figure 3D) genes. The only gene which gave a significant difference was *dhhc-6*, whose knockdown gave a reduction in thrashing rate. Locomotion of all the mutant strains was also assessed on solid medium (Additional file 8, panels A and B). Only the *dhhc-9* (*gk985*) and *ppt-1* (*gk134*) strains showed a significant difference from wild-type locomotion. When an RNAi approach was used, none of the genes tested showed an obvious change in locomotory rate (Additional file 8, panel C).

As palmitoylation is known to be involved in a number of neurodegenerative processes [46–51], a possible readout would be a faster than normal decrease in locomotion rate as *C. elegans* ages. All of the mutant strains except *dhhc-1*, which was not yet available at the time of the experiment, were synchronised and subjected to thrashing assays as they aged (Additional file 9). None of the strains showed a significant difference from the shape of decay in the wild-type N2 rate of thrashing.

It is possible that this lack of strong locomotory phenotypes is due to a certain level of redundancy, that is, that other DHHCs can take over the roles of missing enzymes. Feeding RNAi can be used to knock down expression of multiple genes at the same time, albeit at a reduced efficiency compared with feeding RNAi against only one gene, by culturing the animals on a mixture of bacterial strains (Additional file 10) [25, 52, 53]. In order to test whether redundancy may play a part in locomotion assays, pairs of closely related DHHCs were knocked down based on phylogenetic analysis (Figure 3E). These thrashing assays only showed a difference with knockdown of *dhhc-1* and -*10* together, giving a small but significant increase in speed. The rest of the combinations thrashed at the same rate as the negative control.

It has been observed that knockdown of two or more genes by mixing the feeding RNAi bacterial strains gives variable efficacy in an apparently gene-dependent manner [25, 52, 53]. Recently described is an approach in which the sequences from two or more genes of interest are cloned into the same vector, giving efficient knockdown of all the genes involved [52, 53]. As there are only two known PPTs in *C. elegans*, this approach was tested by comparing the thrashing rates after knockdown of *ppt-1* and *ath-1* either by mixing the individual bacterial strains or by feeding a bacterial strain expressing the conjugated sequences from the individual vectors (Additional file 11). However, as no difference was seen from the negative control in either condition despite apparently more efficient knockdown (Additional file 10), this was not pursued further.

Another phenotype which can occur due to dysfunction of a subset of neurons is a defect in mechanosensation, whereby animals fail to respond to a mechanical stimulus by increasing movement in the opposite direction [33]. Selected mutant strains were tested for the presence of such a phenotype (Additional file 12). All of the strains were found to have normal responses to both anterior and posterior mechanical stimuli.

### Ageing phenotypes

**Figure 4:**
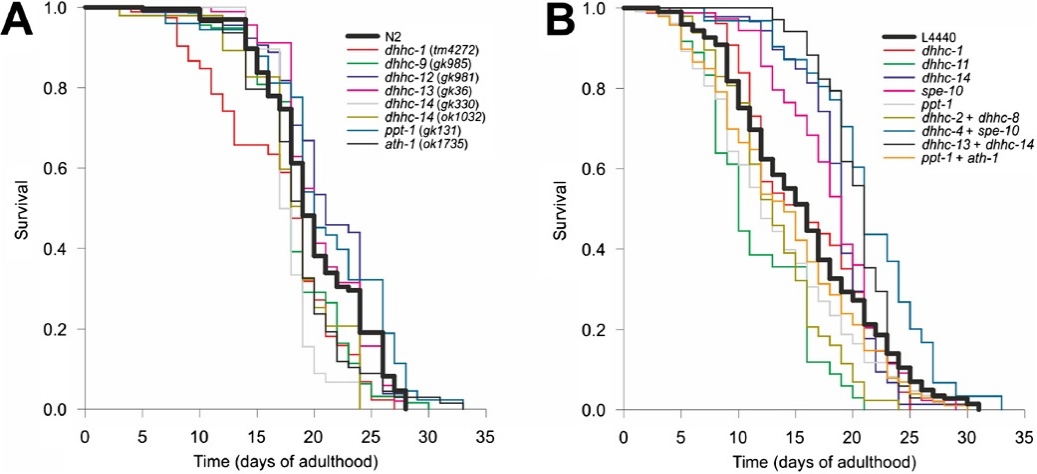
**Disruption of some palmitoylation enzymes causes changes in survival.** Survival plots are shown of mutant strains **(A)** and RNAi experiments **(B)** which differ significantly from the control in at least one measure (see Tables 2 and 3). The control strain was Bristol N2 for mutants and *rrf-3* mutants fed bacteria containing the empty vector (L4440) for RNAi.

The only DHHC enzyme whose loss has been studied previously is SPE-10. One of the observations which has been made is a small increase in lifespan [29]. It was therefore decided to investigate whether any of the other mutant strains obtained also had any differences in lifespan. Values for mean and median lifespan, 90% mortality and maximum lifespan of mutant strains are collated in Table 2. Survival plots of strains showing a significant difference from wild-type N2 animals are shown in Figure 4A, and those with no difference from N2 in Additional file 13, panel A. *dhhc-1*, *dhhc*-*9* and both *dhhc-14* mutants showed a decrease in mean lifespan. *dhhc-14* (*gk330*) mutants also showed a reduced median lifespan, and *dhhc-1* and both *dhhc-14* mutants had reduced time to 90% mortality. The *spe-10* mutant did not show an increased mean or median lifespan as previously reported, although its maximum lifespan was extended considerably compared with wild-type. The *ath-1* mutant also showed decreased time to 90% mortality; an increase in median lifespan was seen in *dhhc-12*, *dhhc-13* and *ppt-1* (*gk131*) mutants but not in other *ppt-1* strains.

**Table 2:** **Survival analysis of DHHC and PPT mutant strains**

Strain	***n***	Mean (days)	Median (days)	90% mortality (days)	Maximum (days)
N2	184	20	19	26	28
*dhhc-1* (*tm4272*)	97	17*	18	24^†^	27
*dhhc-2* (*ok990*)	100	20	19	26	34
*dhhc-9* (*gk985*)	143	19*	19	24	30
*dhhc-12* (*gk1013*)	50	19	20	26	26
*dhhc-12* (*gk981*)	93	21	21^†^	26	28
*dhhc-13* (*gk36*)	93	21	20^†^	26	28
*dhhc-14* (*gk330*)	49	18*	18^†^	20^†^	24
*dhhc-14* (*ok1032*)	48	18*	19	24^†^	24
*spe-10* (*hc104*)	95	21	19	29	35
*ppt-1* (*gk131*)	88	21	20	28^†^	33
*ppt-1* (*gk134*)	43	19	19	23	28
*ppt-1* (*gk139*)	86	19	19	27	30
*ath-1* (*ok1735*)	80	19	19	24^†^	33

Various measures of survival were extracted from lifespan experiments comparing wild-type Bristol N2 and DHHC or PPT mutant strains. Statistical tests were applied to values varying from the control using OASIS [73]. * *p* < 0.01 by the log-rank (Mantel-Cox) test. ^†^ *p* < 0.01 by Fisher’s exact test.

Lifespan analysis was also carried out on *rrf-3* mutants treated with RNAi against genes of interest along with combinatorial RNAi with related enzymes identified by the phylogenetic analysis (Table 3). Survival plots are shown in Figure 4B, for those showing significant changes from the negative control strain, and Additional file 13, panel B, for those which did not. Of the RNAi experiments with single genes, *dhhc-11* showed a decrease in mean lifespan. *dhhc*-*14* and *spe-10* had increased median lifespan, while that of *ppt-1* was decreased. *dhhc-1* and -*14* also showed a decrease in time to 90% mortality which was also seen with their mutant strains, suggesting they may be involved in processes at older age. However, the reduced mean lifespan seen in the mutant strains was not observed with RNAi of *dhhc-1* and -*14.* Combined knockdown of *dhhc-2* with -*8* and *ppt-1* with *ath-1* gave a reduction in mean survival time. In contrast concurrent knockdown of *dhhc-4* and *spe-10* resulted in an increase in mean and median lifespan. The mixture of *dhhc-13* and -*14* also gave an increased median lifespan.

**Table 3:** **Survival analysis of** ***rrf-3*** **mutants treated with RNAi against DHHCs and PPTs**

RNAi strain	***n***	Mean (days)	Median (days)	90% mortality (days)	Maximum (days)
L4440	208	16	16	25	31
*dhhc-1*	79	16	16	24^†^	25
*dhhc-11*	47	12*	10	18	21
*dhhc-14*	99	19	19^†^	22^†^	31
*spe-10*	79	18	19^†^	24	29
*ppt-1*	108	13	12^†^	23	30
*ath-1*	47	16	16	25	26
**RNAi strain(s)**	***n***	**Mean (days)**	**Median (days)**	**90% mortality (days)**	**Maximum (days)**
L4440	208	16	17	25	31
*dhhc-1* + *dhhc-10*	50	18	18	24	29
*dhhc-2 + dhhc-8*	50	13*	13	20^†^	24
*dhhc-3* + *dhhc-7*	23	19	20	26	28
*dhhc-4* + *spe-10*	34	21*	21^†^	27	33
*dhhc-5* + *dhhc-6*	27	17	16	25	26
*dhhc-9* + *dhhc-12*	44	18	18	24	26
*dhhc-13* + *dhhc-14*	38	21	21^†^	24	30
*ppt-1* + *ath-1*	143	14*	14	23	30

Various measures of survival were extracted from lifespan experiments comparing hypersensitive *rrf-3* animals treated with feeding RNAi empty vector L4440 control or against DHHC or PPT mutant strains (top). Mixtures of feeding RNAi bacteria were also used to assess simultaneous knockdown of related enzymes (bottom). Statistical tests were applied to values varying from the control using OASIS [73]. *p < 0.01 by the log-rank (Mantel-Cox) test. ^†^ *p* < 0.01 by Fisher’s exact test.r.

## Discussion

In comparison with other organisms important to research, palmitoylation has been neglected as a field of study in the model organism *C. elegans*. Indeed, there is even confusion about the number of DHHC enzymes encoded in its genome, with figures of 15–17 reported [1, 4, 27]. Here, we have mined the official *C. elegans* genome database, Wormbase, using the conserved DHHC-CRD to conclusively show there are 15 DHHC genes encoded. A systematic naming procedure of *dhhc-x* was applied to the unnamed *C. elegans* genes and their products in this family, except for *spe-10* which has been previously published [27–29]. Sequence analysis confirmed that all of these proteins contained the requisite DHHC-CRD which is the active site [12, 54]. Although palmitoyltransferase activity is yet to be directly observed with these enzymes, their overall structure is in line with enzymes whose activity has been confirmed. DHHC enzymes have a common structure of at least four TMDs, with the DHHC motif found in the cytoplasmic loop between TMD2 and TMD3 [1, 4, 6, 35]. An obvious discrepancy in the *C. elegans* family occurs with DHHC-11, which omits the two TMDs normally found before the DHHC-CRD and is isolated in the phylogenetic analysis. It is possible that its TMDs are missing as a result of a mutation producing a protein truncated at the N-terminus. Alternatively, palmitoylation of one of the cysteine residues upstream of the first TMD could mediate membrane attachment of the N-terminus of the protein, compensating for the lack of TMDs in this region.

A phylogenetic analysis to group enzymes based on their relatedness can give an initial overview of information on the DHHCs within individual organisms and also with a wider perspective by including families from many organisms. A comparison of the *S. cerevisiae* DHHCs with the *H. sapiens* DHHCs showed several broad sub-families [1]. Some sub-families can be explained by the presence of common domains that are not in the majority of the enzymes. For example, the *S. cerevisiae* enzymes Akr1 and Akr2 cluster with the *H. sapiens* enzymes DHHC17/HIP14 and DHHC13/HIP14L. All of these proteins have ankyrin repeat domains N-terminal of their first TMD. A similar analysis was done including the *D. melanogaster* genes with the ankyrin repeat containing proteins clustered within the same group as above [11]. Application of phylogenetic analysis showed that the *C. elegans* DHHCs tend to form closely related pairs, but that these do not necessarily have high homology with other organisms. In addition, analysis of the two *C. elegans* PPTs showed one fell into each of the two known PPT subfamilies.

**Figure 5:**
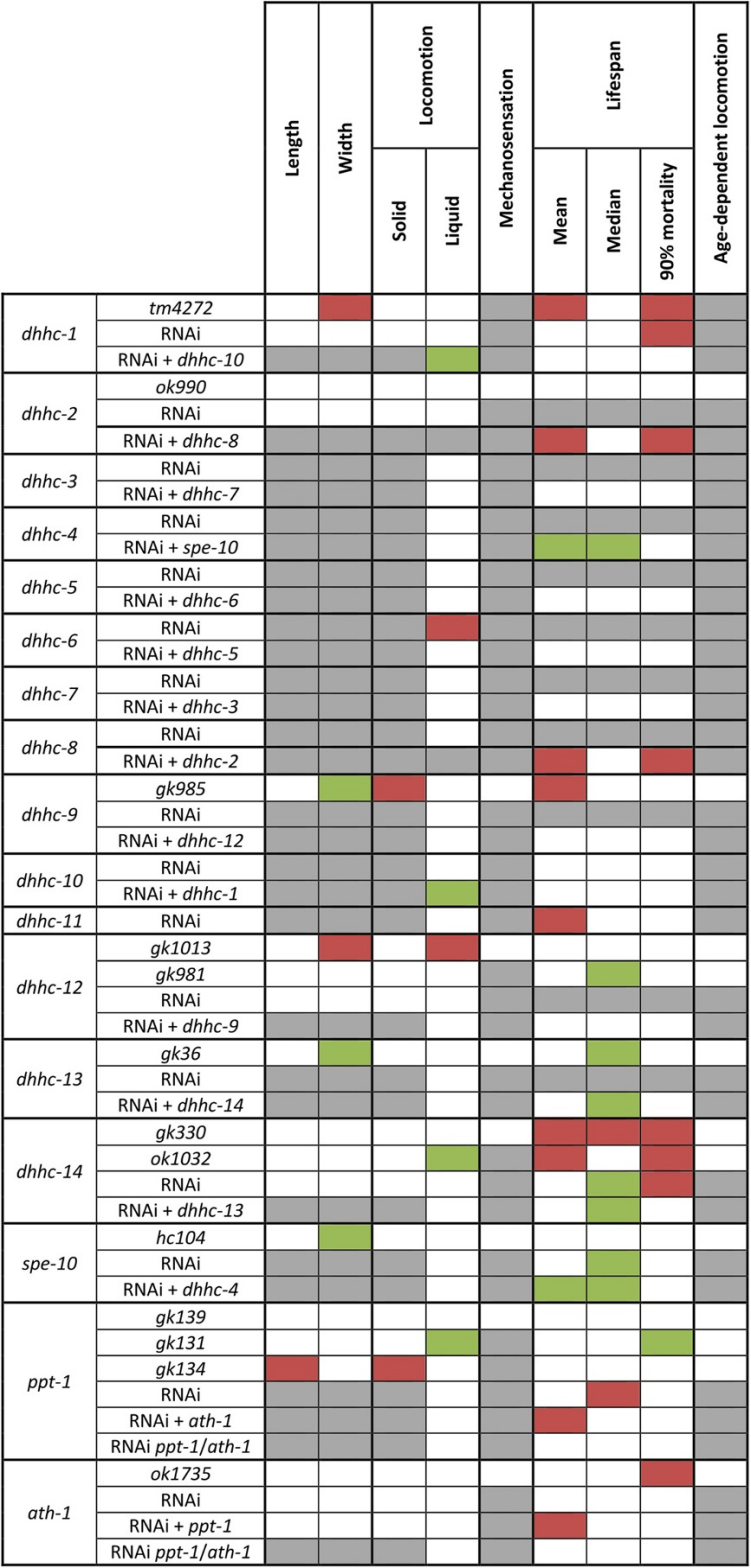
**Summary of observations from the assays performed in this study.** This matrix summarises the results presented in this study. A significant increase in quantity or time is coloured green and a significant decrease is in red. Grey indicates a test was not performed.

To examine the in vivo roles of palmitoylation enzymes a combination of mutant strains and feeding RNAi treatment were used to screen for strong phenotypes associated with their loss, both individually and in combinations. To gain an overview of all the analyses performed, a matrix was produced showing which genes were tested for each assay and the results (Figure 5). A handful of mutants showed slight changes in morphology but this was not seen in any RNAi experiments and is difficult to explain based on current knowledge. It is possible the mutant strains have a mild effect during development which results in morphological differences, or perhaps they retain more or fewer eggs. It is also unclear as to why some phenotypes were seen in mutants but not in RNAi experiments. One possibility is that the remaining low level of protein expression with RNAi is still enough for the enzyme to perform its function (Additional file 10). In addition, although we used the RNAi hypersensitive strain, *rrf-3* [55], it may be that some cell types, such as neurons, are still relatively resistant to RNAi, leading to false negative results for enzymes expressed in those cell types. Alternatively, secondary mutations in the mutant strains could explain phenotypes observed in mutants but not with RNAi. Finally, differences in the molecular composition of the OP50 and HT115 strains used for mutant and RNAi experiments, respectively, could conceivably impact on lipid metabolism and hence palmitoylation within the worm. Although further repeat RNAi experiments could potentially reveal some additional statistically significant effects, these would still represent minor phenotypes. Therefore, although the above caveats need to be emphasized, it seems that mutation/knockdown of individual palmitoylation genes does not result in strong phenotypes in *C. elegans*.

The DHHC SPE-10 has already been studied in relation to its spermatogenesis phenotype [27, 28] and resistance to certain stresses [29]. The *spe-10* mutant strain exhibited reduced fertility, but the moderate increase in lifespan previously reported in some experiments [29] was not observed here (Table 2). However these experiments were performed at 20°C in contrast to the published observations at 25.5°C. As *spe-10* mutants also have a resistance to heat stress [29], it is possible this is the main factor determining lifespan at higher temperatures. Nevertheless an increase in maximum lifespan was observed both in that study and here.

As many of the DHHC enzymes are closely related some other phenotypes may be masked by a compensatory effect where the similar enzyme takes over the function of the disrupted one. As well as this, many substrates can be palmitoylated by more than one palmitoyltransferase [9, 56, 57]. In view of this, some combinatorial RNAi was also performed. Interestingly, some of the pairs of DHHCs which emerged from the phylogenetic analysis also share high homology with the same protein in other organisms. For example DHHC-4 and SPE-10 share homology to *S. cerevisiae* Pfa3, *D. melanogaster* CG1407 and *H. sapiens* DHHC2. This similarity in these pairs suggests that they may share common substrates and functions within the cell. Indeed this is the case with RNAi against both *spe-10* and *dhhc-4* simultaneously, extending the increase in mean and median lifespan seen with knockdown of *spe-10* only (Table 3). Additionally the *dhhc-2* mutant showed no change in lifespan, but when knocked down in combination with *dhhc-8* there was a decrease in mean lifespan and time to 90% mortality. However other combinations did not result in additional phenotypes. It may be that efficient knockdown of many DHHC enzymes simultaneously is required to uncover more severe phenotypes.

The primary aim of the functional part of our systematic study was to identify strong phenotypes caused by mutation/knockdown of palmitoylation genes. Given that mutations in single PATs result in readily observable overt phenotypes in mammals and in flies [50, 51, 58, 59], the lack of strong phenotypes in worms was somewhat unexpected. This may be due, in part, to the technical issues discussed above. Alternatively, it may be that PATs perform more basic cellular housekeeping functions in relatively simple organisms, which might be more readily compensated for, but take on more specialized roles in more complex animals. This notion is supported by the relative difficulty of finding strong phenotypes in *S. cerevisiae,* with knockout of multiple DHHC family members simultaneously often being required [20]. Another possibility is that the functional impact of key palmitoylated protein substrates may differ in divergent organisms. For example, mutation of a *Drosophila* PAT, HIP14, causes a strong impairment of neurotransmission that is entirely due to non-palmitoylation of cysteine string protein (CSP), as expression of a chimaeric CSP artificially targeted to synapses fully rescues this defect [59]. However, the extremely severe phenotype of CSP mutants seen in *Drosophila* [60] is not observed in *C. elegans* null mutants [61], so even if mutation of a worm PAT(s) completely prevented CSP palmitoylation, this would not result in strong effects on the phenotypes analysed in the present study.

The lack of any conserved motifs has limited the known PPTs to two sub-families: PPTs and APTs. Despite this, crystal structures of *H. sapiens* PPT1, PPT2 and APT1 show the same basic structure of an α/β hydrolase fold and a catalytic triad of serine, aspartate and histidine residues [18, 62, 63]. It is surprising that some organisms only have one of these enzymes given the relative abundance of different DHHCs. Analogous reversible post-translational modifications do not have quite this extreme a ratio of enzymes. The human genome contains around 500 kinases [64] compared with approximately 200 phosphatases [65] and in the ubiquitin system there are about 600 E3 ligases [66] and nearly 90 deubiquitinases [67]. It is possible that there are other PPTs which are yet to be discovered, otherwise mutants lacking them would be expected to have more severe phenotypes than are observed. Deletion of the only *S. cerevisiae* enzyme, Apt1, does not lead to any gross phenotype despite its depalmitoylation activity on Gα [68]. Inhibition of APT1 by palmostatin B in Madin-Darby canine kidney epithelial (MDCK) cells prevented N-Ras depalmitoylation and altered its subcellular localisation from the plasma membrane and Golgi to all cellular membranes [69]. Despite this mislocalisation of an important signaling molecule, the cells showed no obvious phenotype. Similarly, a study identifying APT1 as the PPT for BK channels reported no phenotypes despite the channels becoming trapped in the ER [70]. If Apt1 is the only PPT in *S. cerevisiae* and it is responsible for depalmitoylation of substrates such as Gα and Ras then it seems there is some compensatory mechanism which prevents manifestation of a severe phenotype. Redundancy for these palmitoylated client proteins is a likely explanation for how the cell can buffer such effects on key substrates.

Mutations in the human PPT1 gene are known to cause the neurodegenerative disease infantile neuronal ceroid lipofuscinosis (INCL) [31]. *ppt-1* mutants in *C. elegans* show only a mild mitochondrial defect which is considerably less severe than the human INCL symptoms [30]. Analysis of *ppt-1* in this study produced somewhat challenging data from which to draw conclusions. The *gk131* mutant showed an increase in thrashing rate, which was not replicated by RNAi, and the *gk134* allele showed a decrease in locomotion on solid media. Lifespan effects also varied, with the *gk131* strain showing increased mean lifespan and time to 90% mortality, whereas *ppt-1* RNAi showed a decreased median lifespan. Mutants of *ath-1* showed a significant decrease in time to 90% mortality, and in combination with *ppt-1* there was a reduction in mean lifespan. The disruption of both PPTs in the same *C. elegans* strain and the comparison of palmitoyl-proteomes of wild-type and PPT mutants may help shed light on the roles the PPTs play. This would be valuable in expanding the knowledge of PPT substrates and help to answer the question of whether PPTs have distinct sets of substrates or whether there is an overlap.

## Conclusions

This study set out to define the DHHC family of enzymes in *C. elegans*, to characterise both the DHHCs and PPTs and to screen for strong phenotypes associated with disruption of them. As only *spe-10* and *ppt-1* had been previously characterised, the additional phenotypes identified for both these and other genes validate *C. elegans* as a model for studying palmitoylation in a simple in vivo context. *C. elegans* has been neglected as a tool for studying palmitoylation, but these data can be used as a starting point for future studies into specific enzymes, helping to further define the relationship between palmitoylation enzymes and diverse cellular processes.

## Methods

### Database mining

Initial searches for putative *C. elegans* DHHC enzymes were conducted on WormBase (http://www.wormbase.org/) using the Pfam tag of DHHC zinc-finger domain (PF01529). PPTs were found by basic local alignment search tool (BLAST) searching (http://blast.ncbi.nlm.nih.gov/) using the known *H. sapiens* PPT sequences; no characteristic PPT domain is known.

Basic information known about the *H. sapiens*, *D. melanogaster* and *S. cerevisiae* DHHCs and PPTs were found by searching the NCBI databases (http://www.ncbi.nlm.nih.gov/). In addition, the following organism-specific resources were used: *Saccharomyces* Genome Database (http://www.yeastgenome.org/); FlyBase (http://flybase.org/); WormBase.

### Sequence analysis

BLAST searches to identify potential orthologues were conducted using the amino acid sequence of the relevant protein. Sequence alignments were performed using the Clustal Omega online program (http://www.ebi.ac.uk/Tools/msa/clustalo/). Colour highlighting of salient features was manually applied.

The domain structure diagram was constructed principally from information predicted by the InterProScan program (http://www.ebi.ac.uk/interpro/) and the TMpred program (http://www.ch.embnet.org/software/TMPRED_form.html). Information on the PaCCT motif was obtained from a published analysis [36].

### Phylogenetic analysis

To generate phylogenetic trees, alignments were first generated using BioEdit (Isis Pharmaceuticals; v6.0.5) and saved in .phy format for use in the suite of programs Phylip v3.69 (http://evolution.genetics.washington.edu/phylip/getme.html). The .phy file was used as the “infile” for Seqboot. 2000 bootstraps were generated and used as the “infile” for Proml, using no outgroups and set to unrooted tree. 10 jumbles were used if computing power sufficed, otherwise one jumble was used. The “outtree” output file was used as the “intree” file for the Consense program. This final “outtree” file was uploaded to the Interactive Tree of Life server (http://itol.embl.de/index.shtml) [71] and formatted for display.

### Nematode husbandry

All *C. elegans* strains were obtained from the *Caenorhabditis* Genetics Center (CGC; University of Minnesota, Twin Cities, MN, USA) except tm4272, which was obtained from the National Bioresource Project for the Experimental Animal “Nematode *C. elegans*” based in the lab of Dr Shohei Mitani (Tokyo Women’s Medical University, Tokyo, Japan). All strains were verified by PCR analysis.

*C. elegans* were cultured in 60 mm plates on nematode growth medium agar (NGM; 2% (w/v) agar, 0.3% (w/v) NaCl, 0.25% (w/v) peptone, 1 mM CaCl_2_, 5 μg ml^-1^ cholesterol, 25 mM KH_2_PO_4_, 1 mM MgSO_4_) at 20°C, seeded with 30 μl *E. coli* OP50 culture (CGC) as a food source, using standard methods [32].

### RNAi

RNAi feeding experiments were conducted using bacteria from the Vidal *C. elegans* ORF RNAi Library [39] (Source Bioscience, Nottingham, UK) where available, or were cloned in-house. All RNAi feeding bacteria were *E. coli* strain HT115, which lacks the dsRNA-specific RNase III but contains an isopropyl β-D-1-thiogalactopyranoside (IPTG)-inducible T7 RNA polymerase [72], carrying the pG-L4440 vector containing the relevant insert. The negative control vector contained no insert (L4440). *hsp-1* was used as a positive control, as it gives a sterile phenotype [55].

RNAi plates of NGM containing 25 μg ml^-1^ carbenicillin and 1 mM IPTG were poured 4–7 days before seeding with 50 μl of the relevant bacterial strain and kept in the dark. After overnight induction, ten L3-L4 stage *rrf-3* (NL2099) worms were transferred to each plate. Once the next generation of worms had reached young adult stage, three adults were moved onto individual replica plates, allowed to lay eggs and removed the following day. These progeny were used for assays once they had reached adulthood.

RNAi knockdown efficiency was assessed by quantitative PCR (QPCR). Total RNA was extracted from worm pellets using Absolutely RNA Microprep Kit (Agilent, Stockport, UK). 8 μl RNA was mixed with 1 μl 40 μM random hexamers and 1 μl 10 mM dNTPs and incubated at 70°C for 5 minutes then placed on ice. 4 μl 5× RT buffer, 1 μl RNase OUT, 1 μl BioScript (Bioline) and 4 μl H_2_O were added to each reaction and incubated at 25°C for 10 minutes, 42°C for 30 minutes and 85°C for 5 minutes before chilling on ice and storing at -20°C. QPCR was performed in a CFX Connect Real Time PCR Detection System (Bio-Rad). 7.5 μl 2× SYBR mastermix was mixed with 1.5 μl 2.5 μM each primer (Additional file 14), 1 μl cDNA template and 3.5 μl H_2_O and amplified using the following conditions: 95°C (10 minutes); 40 cycles of 95°C (15 s), 60°C (15 s) and 72°C (15 s) with a reading taken at the end of each cycle; 95°C (15 s); a gradient from 65°C to 95°C with 0.5°C increments. The ΔΔC_T_ method was used to analyse expression normalised to *act-1* expression and the expression level of each gene in negative control *rrf-3* mutants fed L4440 bacteria (Additional file 10).

### Cloning

In order to clone the RNAi vectors not present in the library, cDNA was first synthesised. 2 μg total RNA from wild-type worms extracted using an Absolutely RNA Microprep Kit (Agilent) was made up to 16.45 μl with diethylpyrocarbonate-treated (DEPC)-H_2_O. 0.8 μl 50 μM oligo(dT) primer was added and incubated at 70°C for ten minutes, then on ice for two minutes. 5 μl Bioscript reaction buffer, 1.25 μl 10 mM dNTPs, 0.5 μl RNase OUT and 1 μl BioScript (Bioline) were added and reverse transcription performed under the following conditions: 25°C (10 minutes), 42°C (60 minutes), 90°C (2 minutes). 75 μl DEPC-H_2_O was added to the products and stored at -20°C.

Reverse transcriptase PCR was performed using the primers listed in Additional file 15. 5 μl cDNA, 2 μl 10 μM forward primer, 2 μl 10 μM reverse primer, 1 μl 10 mM dNTPs, 10 μl 5× high fidelity or GC-rich-optimised buffer and 0.5 μl Phusion DNA polymerase were made up to a total reaction volume of 50 μl with DEPC-H_2_O. The PCR conditions were as follows: 98°C (2 minutes); 30–35 cycles of 98°C (15 s), 60 or 62°C (30 s), 72°C (15–30 s); 72°C (5 minutes); 4°C store.

Gateway™ cloning was used to shuttle each PCR product into the pG-L4440 vector, with BP clonase™ II and LR clonase™ II (Sigma, Dorset, UK) steps performed as per the manufacturer’s instructions. Successful incorporation into the pG-L4440 vector was verified by transforming into DH5α cells and sequencing the purified DNA (DNA Sequencing & Services, University of Dundee, UK). The vectors were then transformed into HT115 cells for consistency with the Vidal RNAi library.

For the RNAi vector containing sequences for both *ppt-1* and *ath-1*, vectors containing *ppt-1* and *ath-1* individually were purified from overnight bacterial cultures. 20 μl DNA was digested at 37°C in a reaction volume of 25 μl: pG-L4440-*ppt-1* was linearised using *Acc65I*; pG-L4440-*ath-1* was digested with *BsrGI* to obtain the *ath-1* sequence but retaining complementary 5’ overhangs to enable its ligation into the pG-L4440-*ppt-1* vector. 5’-phosphates were removed from linearised pG-L4440-*ppt-1* by incubating with 2 μl shrimp alkaline phosphatase (Promega, Madison, WI, USA) for 15 minutes at 37°C. The products were subjected to agarose gel electrophoresis and the pG-L4440-*ppt-1* and *ath-1* bands were excised and purified into 50 μl using a GenElute™ Gel Extraction Kit (Sigma, Dorset, UK). The ligation reaction was performed with 1 μl pG-L4440-*ppt-1* added to 3 μl *ath-1*, 2 μl 5× T4 ligase buffer, 1 μl T4 DNA ligase and 3 μl H_2_O and incubated at room temperature for one hour. The DNA was transformed into DH5α cells and verified by test digest before transforming into HT115 cells.

### Morphology

Individual worms were placed on a freshly seeded plate and allowed to acclimatise for ten minutes. WormTracker software (http://www.mrc-lmb.cam.ac.uk/wormtracker/; v2.0.3.1) [42, 43] was used to track the worms on a microscope stage controlled by computer through an Optiscan II box (Prior) and collect data via a DinoEye Eyepiece Camera (ANMO Electronics Corporation) over a two minute period. Data were analysed frame-by-frame in Worm Analysis Toolbox software (v1.9) and Microsoft® Office Excel® 2007. Any obvious outlier frames resulting from misanalysis by the software were removed and the mean values from all frames in the video of each worm were used.

### Lifespan

To synchronise a worm strain, a relatively full plate of gravid adults was washed with 3.5 ml sterile H_2_O and collected in a 15 ml Falcon tube. 1.5 ml bleach mixture (two parts commercial bleach to one part 5 M NaOH) was added and the tube vortexed well every two minutes for a total of 10 minutes. The tubes were centrifuged at 1500 rpm for one minute and the supernatant removed. 5 ml sterile H_2_O was added and the tubes vortexed before centrifuging again. The supernatant was removed and the pellet of eggs was transferred to the edge of a seeded plate. These worms were picked onto lifespan plates once they reached adulthood, defined as day 0.

For lifespan analysis of mutants, NGM agar plates were prepared containing 50 μM fluorodeoxyuridine (FUdR), to prevent development of any eggs laid, and seeded with 50 μl OP50. For RNAi lifespan studies, worms were already synchronised as a result of the replica plating procedure. Once they reached adulthood, these worms were transferred to lifespan plates seeded with 50 μl RNAi bacteria but containing no FUdR. All worms were only fed their relevant RNAi bacterial strain throughout the assay, and transferred to fresh plates as necessary.

### Locomotion

Worms were synchronised by allowing a gravid adult to lay eggs on a plate overnight before removing it. Worms were assayed once the progeny reached young adult age. For solid media individual worms were transferred to an unseeded or freshly seeded plate and allowed to acclimatise for five minutes. The number of body bends (complete movement of the head from one side to the other and back again) was observed over a period of two minutes [45]. To assess locomotory ability in solution, the worms were subjected to thrashing assays [44]. 100 μl drops of Dent’s Ringer solution (DRS; 10 mM D-glucose, 10 mM HEPES, 140 mM NaCl, 6 mM KCl, 3 mM CaCl_2_, 1 mM MgCl_2_, pH 7.4) containing 0.1% (w/v) bovine serum albumin (BSA) were put in each well of a 96-well plate. One worm was picked into each well and allowed to equilibrate for at least 5 minutes. The number of thrashes (complete movement of the head from one side to the other and back again) in one minute was recorded.

For age-dependent locomotion, mutants were synchronised and maintained as for lifespan experiments. Every couple of days, ten worms of each strain were subjected to thrashing assays in 30 μl drops of DRS + 0.1% BSA on a dish, then returned to their plate. This was continued until a strain had essentially no movement.

### Mechanosensation

The root end of an eyebrow hair was glued to the end of a toothpick, leaving the tapered end free. Before each assay, this was sterilised by dipping in 70% ethanol and allowed to air dry. The worm to be assayed was transferred to an unseeded plate and allowed 5 minutes to acclimatise. Anterior touch response was tested by gently stroking the eyebrow hair across the body behind the pharynx, and posterior touch response by stroking just forward of the anus. A positive response was defined as stopping movement in the direction of the touch or reversal of direction of movement, or for posterior touch suddenly quicker forward movement [33].

### Statistical analysis

All statistical analyses (with the exception of lifespan and age-dependent locomotion) and all graphs were produced using SigmaPlot (Systat Softare, Inc.; v.12.2.0.45). If two data sets were being directly compared, Student’s *t*-test was used. For comparison of multiple data sets in one analysis, a one-way analysis of variance (ANOVA) was used. Age-dependent thrashing was compared using one-way analysis of covariance (ANCOVA) in Microsoft Office® Excel® 2007.

Lifespan analyses were performed using the Online Application for the Survival Analysis of Lifespan Assays (OASIS; http://sbi.postech.ac.kr/oasis/introduction/) [73]. Mean lifespans were compared using the log-rank (Mantel-Cox) test, and mortality at more specific time points was compared using Fisher’s exact test.

### Availability of supporting data

All supporting data are included as additional files.

## Electronic supplementary material

Additional file 1: **A table showing orthologues of C. elegans DHHC enzymes in S. cerevisiae, D. melanogaster and H. sapiens.** (PDF 435 KB)

Additional file 2: **A table showing collated information available on C. elegans DHHC enzymes.** (PDF 417 KB)

Additional file 3: **An alignment of homologues of the palmitoyl-protein thioestases PPT1 and APT1 in diverse species.** (PDF 3 MB)

Additional file 4: **A phylogenetic tree of PPT enzymes in S. cerevisiae, C. elegans, D. melanogaster and H. sapiens.** (PDF 1 MB)

Additional file 5: **A table showing information about the C. elegans strains used in this study.** (PDF 419 KB)

Additional file 6: **A figure showing measurements of the morphology of DHHC and PPT mutants.** (PDF 1 MB)

Additional file 7: **A figure showing measurements of the morphology of rrf-3 mutants treated with feeding RNAi against DHHCs and PPTs.** (PDF 1 MB)

Additional file 8: **A figure showing locomotion on solid medium of DHHC and PPT mutants and feeding RNAi-treated rrf-3 mutants.** (PDF 1 MB)

Additional file 9: **A figure showing age-dependent declines in locomotion of DHHC and PPT mutant strains.** (PDF 1 MB)

Additional file 10: **A figure comparing RNAi knockdown efficiency between single, two mixed and two combined bacterial strains using quantitative PCR.** (PDF 1 MB)

Additional file 11: **A figure comparing locomotion after different methods of concurrent ppt-1 and ath-1 knockdown by feeding RNAi.** (PDF 1 MB)

Additional file 12: **A table showing mechanosensation data from DHHC and PPT mutant strains.** (PDF 80 KB)

Additional file 13: **A figure showing survival plots of DHHC and PPT mutants and RNAi-treated rrf-3 mutants which do not differ significantly from controls.** (PDF 1 MB)

Additional file 14: **A table listing the primers used for QPCR.** (PDF 309 KB)

Additional file 15: **A table listing the primers used during RNAi vector cloning.** (PDF 337 KB)

## Abbreviations

ANCOVA: Analysis of covariance
ANOVA: Analysis of variance
APT: Acyl-protein thioesterase
BLAST: Basic local alignment search tool
CGC: *Caenorhabditis* Genetics Center
DHHC-CRD: DHHC motif-containing cysteine-rich domain
dsRNA: Double-stranded RNA
GABPI: β-1,4-N-acetylgalactosaminyl-transferase B
HIP: Huntingtin-interacting protein
INCL: Infantile neuronal ceroid lipofuscinosis
OASIS: Online application for survival analysis
PaCCT: Palmitoyltransferase conserved C-terminus
PAT: Palmitoyl acyl-transferase
PPT: Palmitoyl-protein thioesterase
QPCR: Quantitative polymerase chain reaction
RNAi: RNA interference
*spe*: Spermatogenesis-deficient
*tag*: Temporarily-assigned gene
TMD: Transmembrane domain.
