# Supplementary material for: A systematic analysis of protein palmitoylation in Caenorhabditis elegans
Source: BMC Genomics. 2014 Oct 2;15(1):841. doi: 10.1186/1471-2164-15-841 (PMC4192757; doi:10.1186/1471-2164-15-841)
Supplement: Supplementary file 1 — Additional file 1: A table showing orthologues of C. elegans DHHC enzymes in S. cerevisiae, D. melanogaster and H. sapiens. (PDF 435 KB) [file 12864_2014_6518_MOESM1_ESM.pdf]

C. elegans DHHC	*S. cerevisiae*			*D. melanogaster*			*H. sapiens*		
	**Protein**	**E value**	**Identity**	**Protein**	**E value**	**Identity**	**Protein**	**E value**	**Identity**
DHHC-1	Pfa3	8e-14	28%	CG10344B	1e-24	26%	DHHC24	6e-23	32%
DHHC-2	Erf2	3e-30	31%	AppH	7e-86	46%	DHHC14 isoform 2	9e-94	42%
DHHC-3	Pfa3	2e-22	37%	CG8314	2e-86	50%	DHHC3 isoform 1	4e-98	56%
DHHC-4a	Pfa3	2e-20	26%	CG1407A	2e-78	38%	DHHC2	1e-79	38%
DHHC-4b	Pfa3	1e-20	26%	CG1407A	2e-78	38%	DHHC2	7e-80	38%
DHHC-4c	Pfa3	6e-21	26%	CG1407A	1e-75	42%	DHHC2	2e-77	39%
DHHC-4d	Pfa3	2e-23	28%	CG1407A	5e-81	39%	DHHC2	2e-83	40%
DHHC-4e	Frk1*	0.037	25%	CG1407A	1e-20	36%	DHHC2	5e-21	49%
DHHC-4f	Ade5,7*	0.035	27%	CG1407A	1e-09	30%	DHHC2	4e-09	49%
DHHC-5	Pfa3	8e-16	29%	CG8314	4e-20	30%	DHHC21	7e-22	30%
DHHC-6	Pfa4	2e-19	34%	CG5196A	5e-81	36%	DHHC6	1e-86	35%
DHHC-7a	Pfa3	5e-17	35%	Dnz1	7e-80	42%	DHHC7 isoform 2	7e-49	34%
DHHC-7b	Pfa3	8e-18	32%	Dnz1	2e-60	44%	DHHC3 isoform 1	6e-34	35%
DHHC-8	Erf2	4e-23	31%	CG34449D	2e-76	51%	DHHC8 isoform 2	5e-75	50%
DHHC-9	Erf2	3e-17	26%	CG5880	3e-35	29%	DHHC16 isoform 2	1e-31	34%
DHHC-10	Pfa4	6e-15	41%	CG10344B	1e-18	44%	DHHC24	3e-15	31%
DHHC-11	Swf1	1e-15	33%	CG17075A	6e-19	38%	DHHC11	3e-20	28%
DHHC-12	Pfa4	4e-16	42%	CG5196B	1e-17	27%	DHHC16 isoform 2	3e-17	29%
DHHC-13	Akr1	2e-33	28%	Hip14	3e-43	31%	DHHC17	5e-42	28%
DHHC-14a	Akr2	1e-35	24%	Hip14	1e-68	29%	DHHC17	2e-72	30%
DHHC-14b	Swf1	1e-14	31%	AppO	6e-21	27%	DHHC17	6e-25	33%
SPE-10	Pfa3	1e-17	27%	CG1407B	3e-43	31%	DHHC2	3e-37	31%

Additional File 1. Orthologues of *C. elegans* DHHC enzymes in other organisms. BLAST searches were conducted using the protein sequences of each of the *C. elegans* DHHC enzymes. The closest matches in *S. cerevisiae*, *D. melanogaster* and *H. sapiens* are shown along with relevant parameters. Orthologues which are not DHHC family proteins are indicated with an asterisk. Ade, adenine-requiring; Akr, ankyrin repeat-containing; App, approximated; Dnz, DNZDHHC/NEW1 zinc finger protein; Erf, effect on Ras function; Frk, fatty acyl-CoA synthetase and RNA processing-associated kinase; Hip, huntingtin-interacting protein; Pfa, protein fatty acyltransferase; Swf, spore wall formation.
