# Supplementary material for: A systematic analysis of protein palmitoylation in Caenorhabditis elegans
Source: BMC Genomics. 2014 Oct 2;15(1):841. doi: 10.1186/1471-2164-15-841 (PMC4192757; doi:10.1186/1471-2164-15-841)
Supplement: Supplementary file 2 — Additional file 2: A table showing collated information available on C. elegans DHHC enzymes. (PDF 417 KB) [file 12864_2014_6518_MOESM2_ESM.pdf]

Gene	Chromosome	Protein ID(s)	Gene Expression	PaCCT motif	Phenotypes	Other Information
dhhc-1	X	NP_510510.1				
dhhc-2	I	NP_493007.2				
dhhc-3	I	NP_491702.1				
dhhc-4	III	a: NP_001023032.1<br>b: NP_001023033.1<br>c: NP_001122751.1<br>d: NP_001255040.1<br>e: NP_001255041.1<br>f: NP_001255038.1<br>g: NP_001255039.1	ubiquitous			isoforms a-d have the DHHC motif; part of the C-terminal region is in all isoforms
dhhc-5	III	NP_498488.2	excretory cell, intestine (weak)	a.a. 208-223	embryonic/post-embryonic development variant (RNAi)	
dhhc-6	IV	NP_502302.2	head neurons (larva), intestine (adult)		sterile, embryonic lethal, larval arrest, reduced brood size (RNAi)	
dhhc-7	I	a: NP_492960.1<br>b: NP_492961.1				isoform b has an N-terminal truncation before the DHHC region
dhhc-8	III	NP_499713.3		a.a 234-248	embryonic/post-embryonic development variant (RNAi)	predicted protein binding motif
dhhc-9	X	NP_508435.2				
dhhc-10	X	NP_508805.3	ubiquitous, stronger in anterior pharynx			
dhhc-11	I	NP_491675.3	spermathecal valve (L3/L4), head neurons			
dhhc-12	I	NP_492753.2	possibly in two head and one tail neurons			
dhhc-13	IV	NP_500889.1			fat content variant/reduced (RNAi)	
dhhc-14	X	a: NP_001024514.2<br>b: NP_001024515.1	generalised, possibly neuronal			isoform b has an N-terminal truncation before the DHHC region
spe-10	V	NP_001021339.1		weak: a.a. 287-301	transgene induced cosuppression (RNAi); hermaphrodite sterile; unfertilised eggs laid; increased lifespan; increased stress resistance	

Additional File 2. Collated information on *C. elegans* DHHC enzymes. Information was collected from WormBase, the National Center for Biotechnology Information website (http://www.ncbi.nlm.nih.gov) and (Gonzalez Montoro et al., 2009). PaCCT, palmitoyltransferase conserved C-terminus; *spe*, spermatogenesis deficient.
