# Supplementary material for: A systematic analysis of protein palmitoylation in Caenorhabditis elegans
Source: BMC Genomics. 2014 Oct 2;15(1):841. doi: 10.1186/1471-2164-15-841 (PMC4192757; doi:10.1186/1471-2164-15-841)
Supplement: Supplementary file 3 — Additional file 3: An alignment of homologues of the palmitoyl-protein thioestases PPT1 and APT1 in diverse species. (PDF 3 MB) [file 12864_2014_6518_MOESM3_ESM.pdf]

A

```
HS-PPT1       MASPGCLWLLAVALLPWTCASR--------ALQHLDPPAPLPLVIWHGMGDSCCNPLSMG 52
MM-PPT1       MASSCSRRLLAAALLPWCCAAW--------ALGHLDPPSPPPLVIWHGMGDSCCNPMSMG 52
XT-PPT-1      --MAACRALTLTALLLTLCNG-------------KTEADPVPLVIWHGMGDSCCNPFSMG 45
AF-PPT1       ---------MTAALLCFLLAGPVLL--VAGSPVYKSNNDTVPLVLWHGMGDSCCNPLSMG 49
DM-Ppt1       MISICCSRFSCILFLLFLIFSLVLSYIWWSPTKGGTNPEVLPVVLWHGMGDTCCVPFSLG 60
CE-PPT-1      ------MR-----Y-----FPLLLCLLAITTAEFRNATKQVPVVMWHGMGDCCCNPLSMG 44
DD-PPT1       ------------MKILFFIILIINCYL----IQNSNSENIRPVVLWHGLGDSGLDPLTMG 44
                                                       *:*:***:**    *:::*

HS-PPT1       AIKKMVEKKIPGIYVLSLEIGKTLMEDVENSFFLNVNSQVTTVCQALAKDPKLQQGYNAM 112
MM-PPT1       VIKKMVEKEIPGIYVLSLEIGKNMMEDVENSFFLNVNVQVNMVCQILEKDPKLQQGYNAI 112
XT-PPT-1      AVKKMVEEQIPGIYVLSLEIGNSIPEDMKNSFFLNVNEQVDGVCKELAQNPNLKNGYNSM 105
AF-PPT1       SIKKMIEEEVSGIYVLSLMIGKNVVQDTENGFFMDVNEQVSMVCSQLAQDPKLKGGYNAM 109
DM-Ppt1       AIMNLIVEQTKGGYVRSLQIGGNVLIDWQSGFFIHPNEQVDYVCKQLLQDEHLAKGYHAI 120
CE-PPT-1      SVKKLFEEQIPGVYVHSLQLGSSITKDIEHGFYANTNELVYMACIKIKNDPELKNGYNAI 104
DD-PPT1       KLKELIENQLPGVYVKNIAIGDSIAEDSFNSFFKNVNEQLEIVCKMLKEDTNLTSGFNAV 104
               : ::. ::  * ** .: :* .:  *   .*: . *  :  .*  : :: .*  *:.::

HS-PPT1       GFSQGGQFLRAVAQRCPSPPMINLISVGGQHQGVFGLPRCPGESSHICDFIRKTLNAGAY 172
MM-PPT1       GFSQGGQFLRAVAQRCPTPPMMTLISVGGQHQGVFGLPRCPGESSHICDFIRKSLNAGAY 172
XT-PPT-1      GFSQGGQFLRAVAQRCPSPPMKNLISIGGQHQGVYGFPRCPGEISHMCDLIRKLLNYGAY 165
AF-PPT1       GFSQGGQFLRAVAQRCPSPPMKNLISIGGQHQGVYGLPRCPGESSNICDMIRKALNNGAY 169
DM-Ppt1       GFSQGGQFLRAVAERCPNPPMRNLITLGGQHQGIFGLPMCPTLTEKPCDYITRLLDNAAY 180
CE-PPT-1      GFSQGAQFLRAVAQRCPNPPMKNLVSVGGQHQGVFGAPYCIGDN-IMCNGVRRLIDLGAY 163
DD-PPT1       GLSQGGQFLRGYVERCNDPPVYNLISLGGQHQGVSSLPRCTALNSTLCKIADDLVELGVY 164
              *:***.****. .:**  **: .*:::******: . * *       *.     :: ..*

HS-PPT1       SKVVQERLVQAEYWHDPIKEDVYRNHSIFLADINQERG-INESYKKNLMALKKFVMVKFL 231
MM-PPT1       SKLVQERLVQAQYWHDPIKESVYRNYSIFLADINQERC-VNESYKKNLMALKKFVMVKFF 231
XT-PPT-1      TREVQDYLVQAEYWHDPINEEQYRNGSIFLADINQERH-VNVSYKQNLQLLEKFVMVKFL 224
AF-PPT1       TDLVQKHLVQAQYWHDPLNDGLYKKHSLFLADINQERA-VNETYRKNLQLLDKFVMVKFL 228
DM-Ppt1       APEVQKALVQATYWHDPIMENKYRLGSTFLADINNELF-INKFYIENLQKLKKFVMVQFL 239
CE-PPT-1      LPFVQKRVVQAQYWHDPNQVEEYKKRSIFLADINNENN-NNPTYKRNLLSLKNLVLVKFN 222
DD-PPT1       EKYVQNTLVPGEYWQDPFNYDEYVEKSVFLADINNVRSEKNQTYKDNLLSVNNFILGEFL 224
                 **. :* . **:**     *   * ******:     *  *  **  :.:::: :*

HS-PPT1       NDSIVDPVDSEWFGFYRSGQAKETIPLQETSLYTQDRLGLKEMDNAGQLVFLATEGDHLQ 291
MM-PPT1       NDSIVDPVDSEWFGFYRSGQAKETIPLQESTLYTEDRLGLKKMDKAGKLVFLAKEGDHLQ 291
XT-PPT-1      NDTMVDPPDTEWFGFYRPGQSKETITLQESNLYMEDRLGLKEMEKAGRLVFLGVEGNHLQ 284
AF-PPT1       QDTVVDPVDTEWFGFLKTGQAKETETLQESVLYKEDRLGLAAMDKAGKLAFLASQGDHLQ 288
DM-Ppt1       NDTIVQPKESQWFQYYTTGQNKVIQPFTESKVYQ--DLGLDKMHRQGQLVFLGVEGDHLA 297
CE-PPT-1      QDHMVVPKDSSWFGFYKDGDIDTILPMNETDLYKEDRIGLKKLHESGRIHFMDVDGDHLQ 282
DD-PPT1       ADTIVVPRESELFGYYQPGQDNIILPMEKTDLYIEDWIGIQQLDNEGRLIKISCPGNHLQ 284
               * :* * ::. * :   *: .    : :: :*    :*:  :.. *::  :   *:**

HS-PPT1       LSEEWFYAHIIPFLG---- 306
MM-PPT1       ISKEWFTAHIIPFLK---- 306
XT-PPT-1      FSKEWFNKNIIPFLH---- 299
AF-PPT1       FTREWFHANLLPYLH---- 303
DM-Ppt1       ISKAWFIQNIVPLLLEK-- 314
CE-PPT-1      IPRSVLVNDIIKKYFM--- 298
DD-PPT1       FTDQWFNQNVIPYLNNQIN 303
              :    :  .::
```

B

```
HS-APT1       ----------------------------MCGNNMSTPLPAIVPAARKATAAVIFLHGLGD 32
MM-APT1       ----------------------------MCGNNMSAPMPAVVPAARKATAAVIFLHGLGD 32
SS-APT1       ----------------------------MCGNSMSVPLPAIIPAARKATAAVIFLHGLGD 32
DM-Apt1       -----------------------------------MAAPVIVEATVKQTATLIFMHGLGD 25
CE-ATH-1      ------------------------------MSAVSNGNPSIVSPRGEHKGTLIFLHGLGD 30
SC-APT1       -----------------------------------MNGLRVAAKIQPARQTIIFLHGLGD 25
ZM-APT-1      MSSLIRLLFTLAAAIGAASLLVASLRRRAPPPGLPAKLVPSSHMAGRNRSFVLWLHGLGD 60
                                                                 ::::*****

HS-APT1       TGHGWAEAF------AGIRSSHIKYICPHAPVRPVTLNMNVAMPSWFDIIGLSP--DSQE 84
MM-APT1       TGHGWAEAF------AGIKSPHIKYICPHAPVMPVTLNMNMAMPSWFDIVGLSP--DSQE 84
SS-APT1       TGHGWAEAF------AGIRTPHVKYICPHAPIKPVTLNMGMSMPSWFDIIGLQT--DAEE 84
DM-Apt1       TGHGWSSAL------AAIRPPFMKVICPTAPTQPVSLNAGFRMPSWFDLKTLDI--GGPE 77
CE-ATH-1      QGHGWADAFK-----TEAKHDNIKFICPHSSERPVTLNMGMRMPAWFDLFGLDP--NAQE 83
SC-APT1       TGSGWGFLAQYLQQRDPAAFQHTNFVFPNAPELHVTANGGALMPAWFDILEWDPS-FSKV 84
ZM-APT-1      SGPANEPIRN---LFSAPEFRLAKWSFPSAPRAPVSCNNGFVMPSWFDIHELPMSAGSPQ 117
               * .       :           :   * :    *: *    **:***:     .  .

HS-APT1       DESGIKQAAENIKALIDQEVKNGIPSNRIILGGFSQGGALSLYTALTTQQKLAGVTALSC 144
MM-APT1       DESGIKQAAETVKALIDQEVKNGIPSNRIILGGFSQGGALSLYTALTTQQKLAGVTALSC 144
SS-APT1       DEAGIKQASENIKALIDQEVKNGIPSHRIVLGGFSQGGALSLYTALTTQQKLGGVVALSC 144
DM-Apt1       DEPGIQSARDSVHGMIQKEISAGIPANRIVLGGFSQGGALALYSALTYDQPLAGVVALSC 137
CE-ATH-1      DEQGINRATQYVHQLIDAEVAAGIPASRIAVGGFSMGGALAIYAGLTYPQKLGGIVGLSS 143
SC-APT1       DSDGFMNSLNSIEKTVKQEIDKGIKPEQIIIGGFSQGAALALATSVTLPWKIGGIVALSG 144
ZM-APT-1      DETGVLKAVEKVHAMIDREVADGIHPENIFVCGFSQGGALTLASVLLYPKKLGGGAVFSG 177
              *. *.  : : :.  :. *:  **   .* : *** *.**:: : :     :.* . :*

HS-APT1       WLPLRASFPQGPIGGANRDISILQCHGDCDPLVPLMFGSLTVEKLKTLVNPANVTFKTYE 204
MM-APT1       WLPLRASFSQGPINSANRDISVLQCHGDCDPLVPLMFGSLTVERLKALINPANVTFKIYE 204
SS-APT1       WLPLRNSFPQASRNSANNEMHVLQCHGEADPLVPVMFGCLTVEKLKTLCNPSNIIFKTYP 204
DM-Apt1       WLPLHKQFPGAKVNSD--DVPIFQAHGDYDPVVPYKFGQLSASLLKSFMK--NVTFKTYS 193
CE-ATH-1      FFLQRTKFPGS--FTANNATPIFLGHGTDDFLVPLQFGQMSEQYIKKFNP--KVELHTYR 199
SC-APT1       FCSIPG-ILKQHKNGINVKTPIFHGHGDMDPVVPIGLGIKAKQFYQDSCEIQNYEFKVYK 203
ZM-APT-1      WVPFSSSVTERISPEAN-KTPFLWSHGMADKVVLFEAGQAGPPFLQSAGV--NCEFKAYP 234
              :      .             .:  **  * :*    *       :      :  :: *

HS-APT1       GMMHSSCQQEMMDVKQFIDKLLPPID---- 230
MM-APT1       GMMHSSCQQEMMDVKHFIDKLLPPID---- 230
SS-APT1       RMPHSACPEEMMDIKQFIEKQLPPI----- 229
DM-Apt1       GLSHSSSDDEMDDVKDIISKWVN------- 216
CE-ATH-1      GMQHSSCGEEMRDVKTFLSAHIAH------ 223
SC-APT1       GMAHSTVPDELEDLASFIKKSLSS------ 227
ZM-APT-1      DLGHSLSQEELLYLESWIKSRLNASQEKDG 264
              : **    :*:  :    :.  :
```

Additional File 3. Alignment of homologues of PPT1 and APT1 in diverse species. These alignments show short regions of homology between the PPT1 (A) and APT1 (B) sequences of different species amongst more divergent stretches of sequence. The catalytic triad [57, 58] is shaded in grey. Absolutely conserved residues are indicated by an asterisk, a colon indicates residues with highly similar properties and a period indicates residues with less similar properties.

AF, *Anoplopoma fimbria*; CE, *Caenorhabditis elegans*; DD, *Dictyostelium discoideum*; DM, *Drosophila melanogaster*; HS, *Homo sapiens*; MM, *Mus musculus*; SC, *Saccharomyces cerevisiae*; SS, *Salmo salar*; XT, *Xenopus tropicalis;* ZM, *Zea mays*.
