# Supplementary material for: A systematic analysis of protein palmitoylation in Caenorhabditis elegans
Source: BMC Genomics. 2014 Oct 2;15(1):841. doi: 10.1186/1471-2164-15-841 (PMC4192757; doi:10.1186/1471-2164-15-841)
Supplement: Supplementary file 4 — Additional file 4: A phylogenetic tree of PPT enzymes in S. cerevisiae, C. elegans, D. melanogaster and H. sapiens. (PDF 1 MB) [file 12864_2014_6518_MOESM4_ESM.pdf]

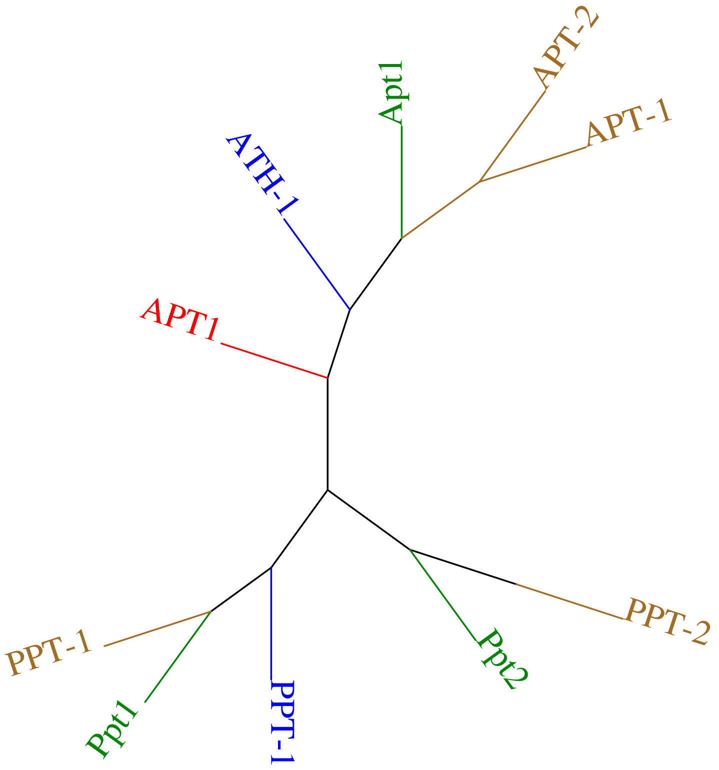

Additional File 4. Phylogenetic tree of PPT enzymes in yeast, worms, fruit flies and humans. The complete sequences of the known PPT family enzymes in *S. cerevisiae* (red), *C. elegans* (blue), *D. melanogaster* (green) and *H. sapiens* (brown) were subjected to phylogenetic analysis. The resulting tree was rendered and coloured using Interactive Tree of Life [66].
