# Supplementary material for: A systematic analysis of protein palmitoylation in Caenorhabditis elegans
Source: BMC Genomics. 2014 Oct 2;15(1):841. doi: 10.1186/1471-2164-15-841 (PMC4192757; doi:10.1186/1471-2164-15-841)
Supplement: Supplementary file 5 — Additional file 5: A table showing information about the C. elegans strains used in this study. (PDF 419 KB) [file 12864_2014_6518_MOESM5_ESM.pdf]

Gene	Strain	Allele	Mutation	Genomic Location	Effect on protein	Outcrossed	Known Phenotypes
ath-1	RB1484	*ok1735*	insertion/ deletion	TAGAGA insertion at -226, then deletion to 1712	Δ1-219 (223)	no	
dhhc-1	tm4272	*tm4272*	deletion	1329-1832	Δ163-241; frame shift from 242 (296)	no	
dhhc-2	RB1044	*ok990*	deletion	10576-11465	Δ230-374; frame shift from 375 (404)	no	
dhhc-9	VC2067	*gk985*	deletion	858-1295	Δ65-83 (311)	no	
dhhc-12	VC2039	*gk1013*	insertion/ deletion	41bp insertion at 1417, then deletion to 1791	Δ216-310 (310)	no	
dhhc-12	VC2244	*gk981*	deletion	717-1321	Δ88-195; frame shift from 196 (310)	x1	
dhhc-13	VC108	*gk36*	deletion	106-446	Δ36-130; frame shift from 131 (782)	no	
dhhc-14	VC771	*gk330*	deletion	1518-2309	Δ243-366 (565)	no	
dhhc-14	VC918	*ok1032*	insertion/ deletion	18bp insertion at 1842, then deletion to 3002	Δ335-494; GRSHLN insertion at 494; frame shift from 495 (565)	no	
ppt-1	MN1	*gk139*	deletion	336-953	Δ84-200 (298)	x4	delay in L4/adult transition; abnormal mitochondrial morphology
ppt-1	VC166	*gk131*	insertion/ deletion	TTT insertion at -22, then deletion to 226	Δ20-100; V insertion at 100 (298)	no	
ppt-1	VC168	*gk134*	deletion	-169-940	Δ33-169; frame shift from 160 (298)	no	
spe-10	BA744	*hc104*	point mutation	C583T	R115* (351)	unclear	defective spermatogenesis; stress resistance

Additional File 5. Information about *C. elegans* strains used in this study. Strain information was collated from WormBase (http://www.wormbase.org/). Genomic location of each mutation refers to nucleotide position in relation to the start of the gene as indicated on WormBase. Effects on protein refer to residue number, with the length of the wild-type protein indicated in parentheses.
