# Supplementary material for: A systematic analysis of protein palmitoylation in Caenorhabditis elegans
Source: BMC Genomics. 2014 Oct 2;15(1):841. doi: 10.1186/1471-2164-15-841 (PMC4192757; doi:10.1186/1471-2164-15-841)
Supplement: Supplementary file 6 — Additional file 6: A figure showing measurements of the morphology of DHHC and PPT mutants. (PDF 1 MB) [file 12864_2014_6518_MOESM6_ESM.pdf]

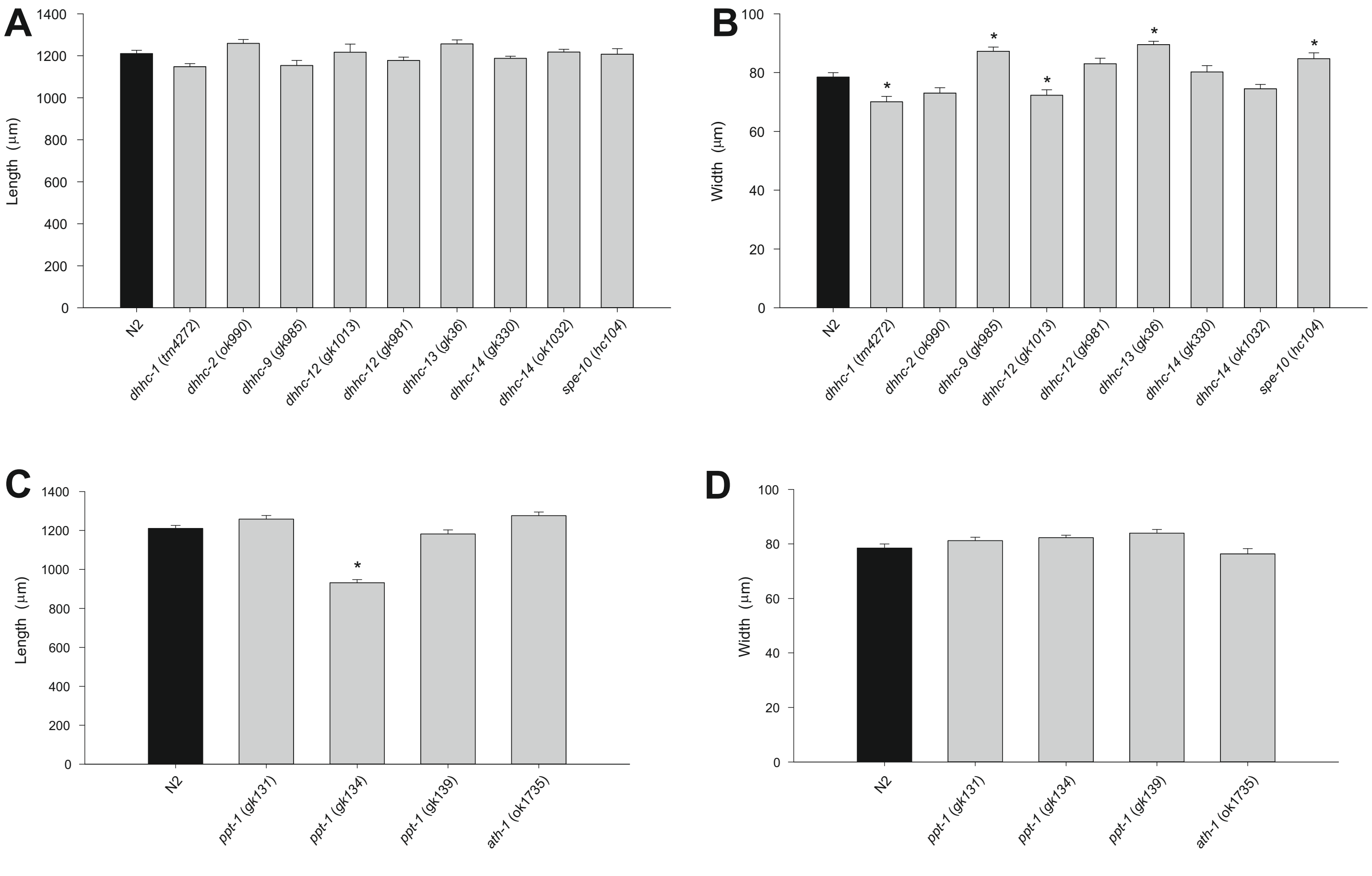

Additional File 6. Some mutants show subtle morphological phenotypes. The length and width of mutants lacking a DHHC (A, B) or PPT (C, D) enzyme along with wild-type Bristol N2 strain were measured using WormTracker software. n = 4-10 animals per strain; * $p < 0.05$ by one-way ANOVA.
