# Supplementary material for: A systematic analysis of protein palmitoylation in Caenorhabditis elegans
Source: BMC Genomics. 2014 Oct 2;15(1):841. doi: 10.1186/1471-2164-15-841 (PMC4192757; doi:10.1186/1471-2164-15-841)
Supplement: Supplementary file 7 — Additional file 7: A figure showing measurements of the morphology of rrf-3 mutants treated with feeding RNAi against DHHCs and PPTs. (PDF 1 MB) [file 12864_2014_6518_MOESM7_ESM.pdf]

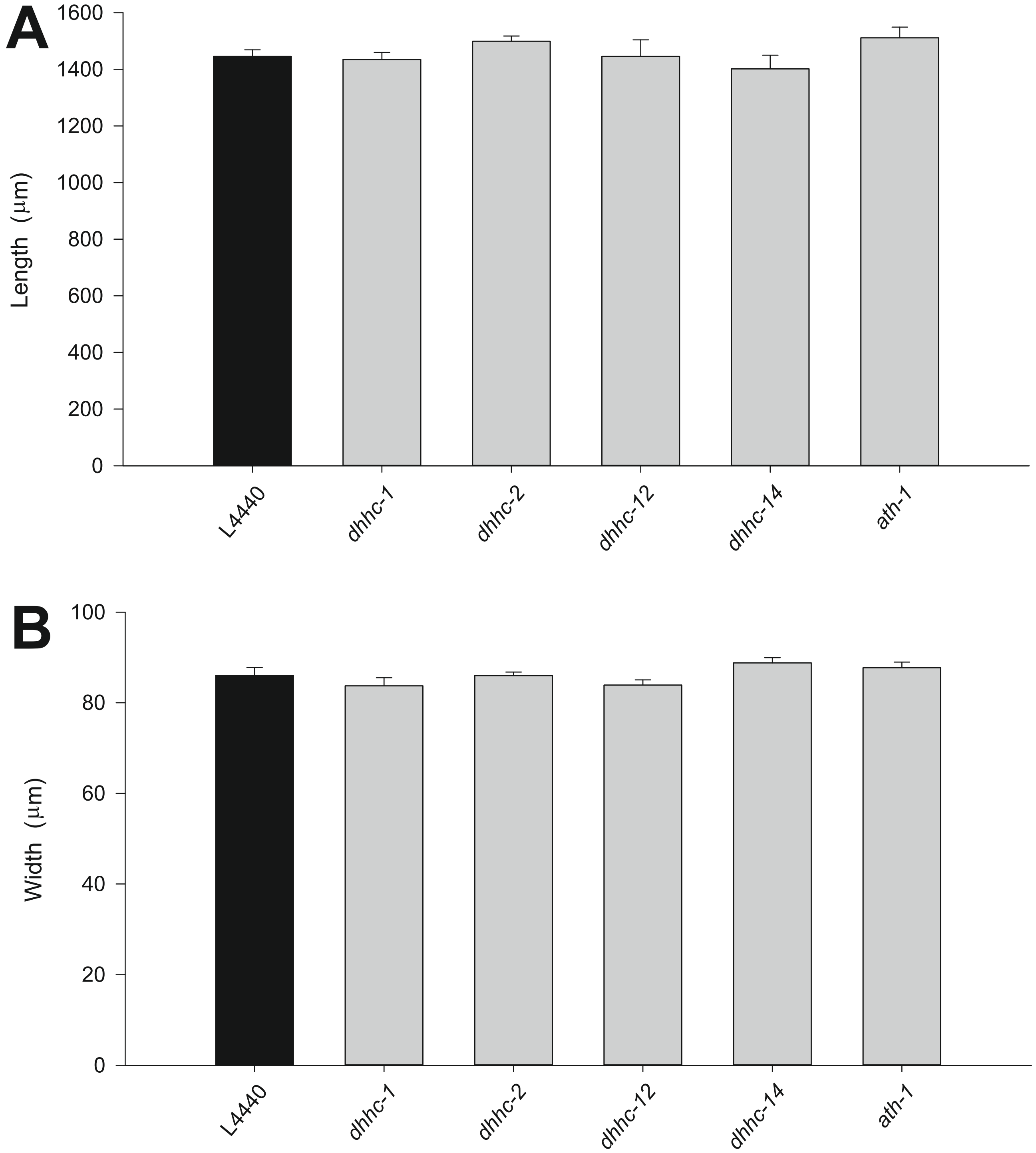

Additional File 7. Feeding RNAi-treated worms show no differences in morphology. *rrf-3* mutant animals were cultured with bacteria expressing dsRNA against a DHHC or PPT enzyme or a negative control empty vector (L4440). Length (A) and width (B) were measured using WormTracker software. No significant differences were found using one-way ANOVA. n = 4-8 animals per RNAi treatment.
