# Supplementary material for: A systematic analysis of protein palmitoylation in Caenorhabditis elegans
Source: BMC Genomics. 2014 Oct 2;15(1):841. doi: 10.1186/1471-2164-15-841 (PMC4192757; doi:10.1186/1471-2164-15-841)
Supplement: Supplementary file 8 — Additional file 8: A figure showing locomotion on solid medium of DHHC and PPT mutants and feeding RNAi-treated rrf-3 mutants. (PDF 1 MB) [file 12864_2014_6518_MOESM8_ESM.pdf]

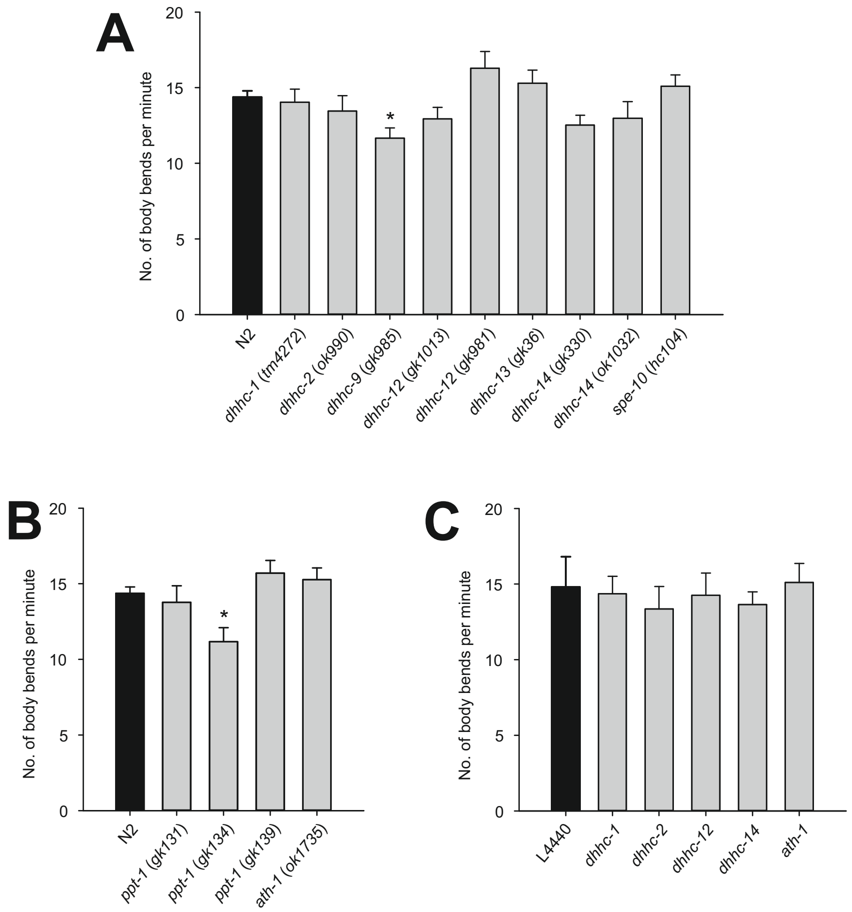

Additional File 8. Locomotive behaviour on solid medium is mostly unaffected by disruption of some palmitoylation enzymes. Locomotion was assessed on solid medium for available mutants of DHHC (A) and PPT (B) enzymes and by feeding RNAi in hypersensitive *rrf-3* strain for a subset of enzymes (C). n = 30-84, 3-12 experiments (mutants) or 5-10, 1 experiment (RNAi). * $p < 0.05$ by one-way ANOVA.
