# Supplementary material for: A systematic analysis of protein palmitoylation in Caenorhabditis elegans
Source: BMC Genomics. 2014 Oct 2;15(1):841. doi: 10.1186/1471-2164-15-841 (PMC4192757; doi:10.1186/1471-2164-15-841)
Supplement: Supplementary file 9 — Additional file 9: A figure showing age-dependent declines in locomotion of DHHC and PPT mutant strains. (PDF 1 MB) [file 12864_2014_6518_MOESM9_ESM.pdf]

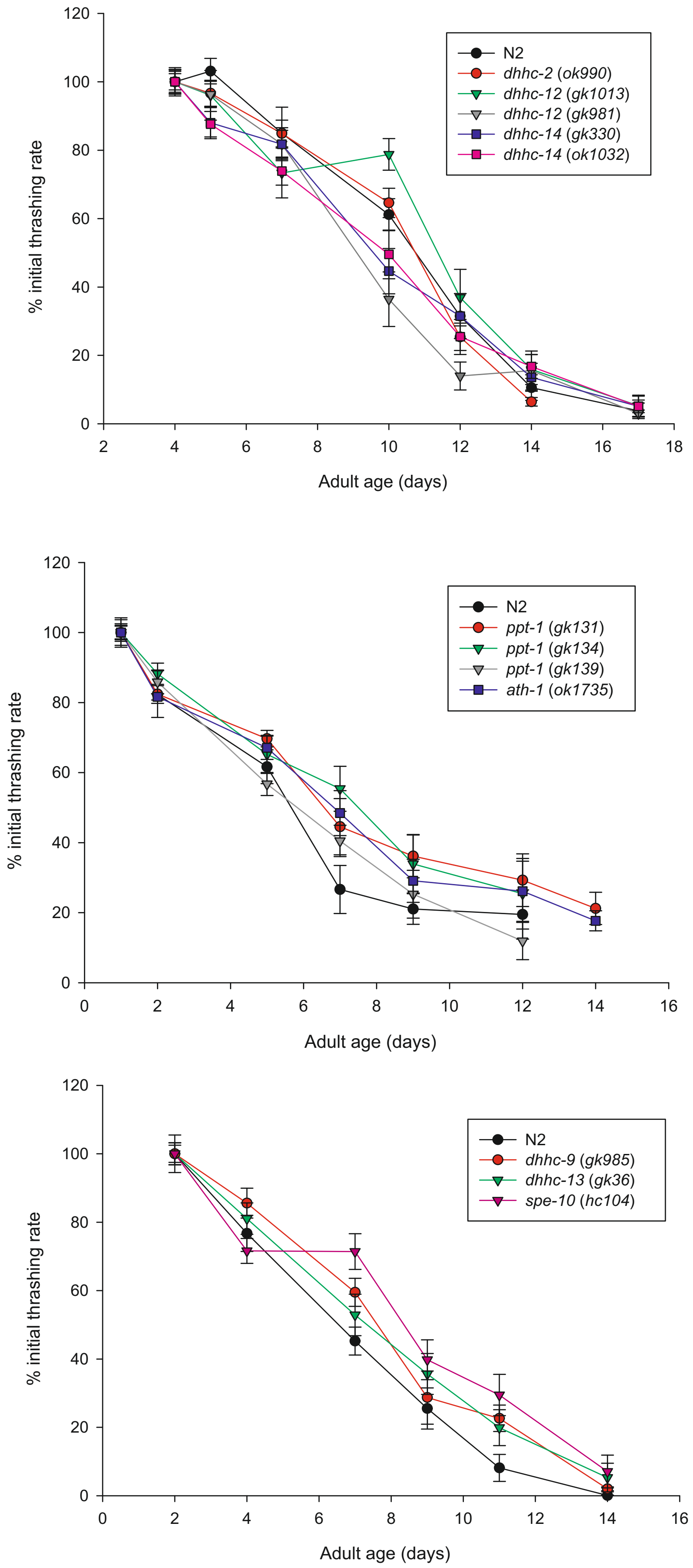

Additional File 9. Age-dependent locomotion of DHHC and PPT mutant strains. Thrashing assays were performed on synchronised animals at various ages and compared with the wild-type N2 strain. Each individual experiment is shown as a separate panel for ease of viewing. No significant difference in decline of locomotion from wild-type was observed in any experiment as assessed by one-way ANCOVA. n = 4-10 animals per strain per time point. ANCOVA, analysis of covariance.
