# Supplementary material for: A systematic analysis of protein palmitoylation in Caenorhabditis elegans
Source: BMC Genomics. 2014 Oct 2;15(1):841. doi: 10.1186/1471-2164-15-841 (PMC4192757; doi:10.1186/1471-2164-15-841)
Supplement: Supplementary file 10 — Additional file 10: A figure comparing RNAi knockdown efficiency between single, two mixed and two combined bacterial strains using quantitative PCR. (PDF 1 MB) [file 12864_2014_6518_MOESM10_ESM.pdf]

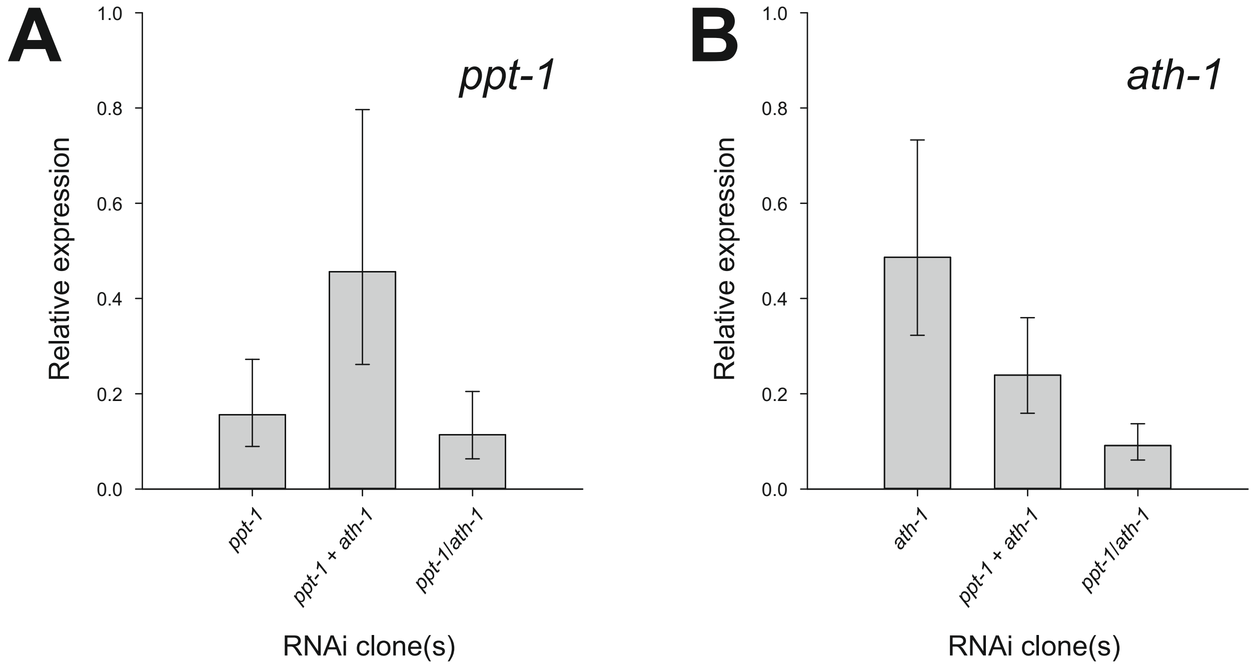

Additional File 10. Assessment of RNAi knockdown efficiency. Total RNA was extracted from worm pellets from plates under three different types of RNAi condition: single clones of *ppt-1* and *ath-1*; a mixture of the clones for *ppt-1* and *ath-1* (*ppt-1* + *ath-1*); and a single clone expressing dsRNA against both *ppt-1* and *ath-1* (*ppt-1*/*ath-1*). cDNA was synthesised and subjected to quantitative PCR. The expression levels of *ppt-1* (A) and *ath-1* (B) were calculated relative to their expression in negative control worms and *act-1* expression using the $\Delta\Delta C_T$ method. The bars represent three technical replicates ± standard deviation.
