# Supplementary material for: A systematic analysis of protein palmitoylation in Caenorhabditis elegans
Source: BMC Genomics. 2014 Oct 2;15(1):841. doi: 10.1186/1471-2164-15-841 (PMC4192757; doi:10.1186/1471-2164-15-841)
Supplement: Supplementary file 11 — Additional file 11: A figure comparing locomotion after different methods of concurrent ppt-1 and ath-1 knockdown by feeding RNAi. (PDF 1 MB) [file 12864_2014_6518_MOESM11_ESM.pdf]

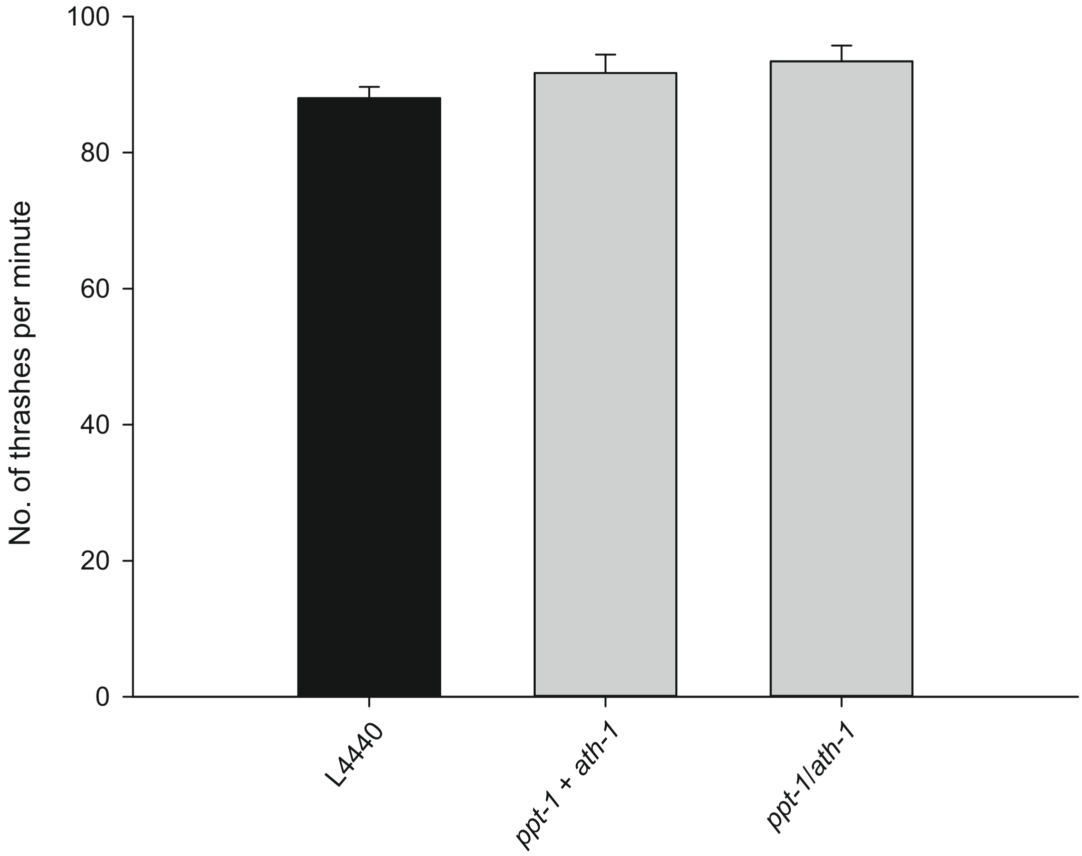

Additional File 11. Different methods of concurrent *ppt-1* and *ath-1* knockdown by feeding RNAi show no locomotion phenotype. *rrf-3* mutant worms were fed either a mixture of individual bacterial strains expressing dsRNA against *ppt-1* and *ath-1* (*ppt-1* + *ath-1*) or a single bacterial strain expressing both dsRNAs (*ppt-1*/*ath-1*). Thrashing in solution was compared with the empty vector control bacteria (L4440). No significant differences in locomotion were observed. *n* = 10-30 worms over 1-3 experiments.
