# Supplementary material for: A systematic analysis of protein palmitoylation in Caenorhabditis elegans
Source: BMC Genomics. 2014 Oct 2;15(1):841. doi: 10.1186/1471-2164-15-841 (PMC4192757; doi:10.1186/1471-2164-15-841)
Supplement: Supplementary file 12 — Additional file 12: A table showing mechanosensation data from DHHC and PPT mutant strains. (PDF 80 KB) [file 12864_2014_6518_MOESM12_ESM.pdf]

Strain	% Response to anterior touch	% Response to posterior touch
N2	97.5	97.5
dhhc-2 (*ok990*)	100	95
dhhc-9 (*gk985*)	100	100
dhhc-12 (*gk981*)	100	100
dhhc-13 (*gk36*)	100	100
dhhc-14 (*gk330*)	100	100
spe-10 (*hc104*)	100	100
ppt-1 (*gk139*)	100	95
ath-1 (*ok1735*)	100	100

Additional File 12. Mechanosensation of DHHC and PPT mutant strains. Mechanosensation was assessed by brushing an eyebrow hair gently over either the anterior or posterior of the animal. A positive response to anterior touch was defined as a change of direction of movement of the animal from forwards to reverse. A positive response to posterior touch was defined as a sudden increase in forward movement. *n* = 20 per strain.
