# Supplementary material for: A systematic analysis of protein palmitoylation in Caenorhabditis elegans
Source: BMC Genomics. 2014 Oct 2;15(1):841. doi: 10.1186/1471-2164-15-841 (PMC4192757; doi:10.1186/1471-2164-15-841)
Supplement: Supplementary file 13 — Additional file 13: A figure showing survival plots of DHHC and PPT mutants and RNAi-treated rrf-3 mutants which do not differ significantly from controls. (PDF 1 MB) [file 12864_2014_6518_MOESM13_ESM.pdf]

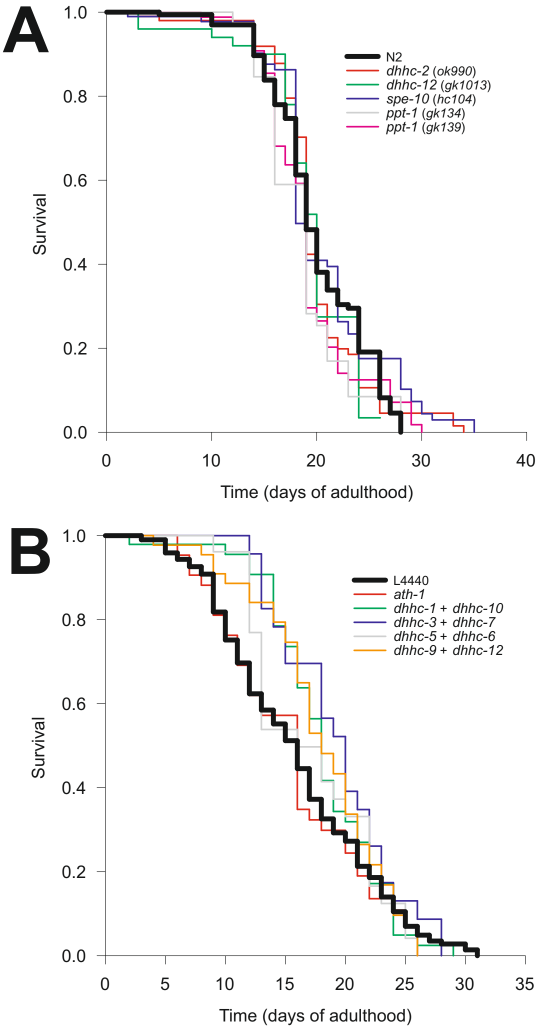

Additional File 13. Survival plots of mutants and RNAi which do not differ significantly from controls. Survival plots are shown of mutant strains (A) and RNAi experiments (B) which do not differ significantly from the control in at least one measure (see Tables 2 and 3). The control strain was Bristol N2 for mutants and *rrf-3* mutants fed bacteria containing the empty vector (L4440) for RNAi.
