# Supplementary material for: A systematic analysis of protein palmitoylation in Caenorhabditis elegans
Source: BMC Genomics. 2014 Oct 2;15(1):841. doi: 10.1186/1471-2164-15-841 (PMC4192757; doi:10.1186/1471-2164-15-841)
Supplement: Supplementary file 14 — Additional file 14: A table listing the primers used for QPCR. (PDF 309 KB) [file 12864_2014_6518_MOESM14_ESM.pdf]

Primer name	Primer sequence
ppt-1 QPCR Fwd	5'-GGAGCACAGTTTTTGAGAGCG-3'
ppt-1 QPCR Rev	5'-TGTTGACCACCGACTGACAC-3'
ath-1 QPCR Fwd	5'-GACTCGGTGATCAAGGGCAT-3'
ath-1 QPCR Rev	5'-GACGTTCAGAGCTGTGTGGA-3'

Additional File 14. Primers for QPCR. The sequences of the primers used for quantitative PCR analysis of RNAi knockdown efficiency are shown.
