# Supplementary material for: A systematic analysis of protein palmitoylation in Caenorhabditis elegans
Source: BMC Genomics. 2014 Oct 2;15(1):841. doi: 10.1186/1471-2164-15-841 (PMC4192757; doi:10.1186/1471-2164-15-841)
Supplement: Supplementary file 15 — Additional file 15: A table listing the primers used during RNAi vector cloning. (PDF 337 KB) [file 12864_2014_6518_MOESM15_ESM.pdf]

Primer name	Primer sequence
dhhc-3 Fwd	5′-***GGGGACAAGTTTGTACAAAAAAGCAGGCT***TGGTCACTTGGTTGCTGGTATC-3′
dhhc-3 Rev	5′-***GGGGACCACTTTGTACAAGAAAGCTGGG***CAAACAGAATACTCGAAAGTTGGTCTC-3′
dhhc-4 Fwd	5′-***GGGGACAAGTTTGTACAAAAAAGCAGGCT***TGTCTTGTCGGGATTGCACAAG-3′
dhhc-4 Rev	5′-***GGGGACCACTTTGTACAAGAAAGCTGGG***TTACGCCGTCACCGAGTCATGTC-3′
dhhc-6 Fwd	5′-***GGGGACAAGTTTGTACAAAAAAGCAGGCT***GGATTGGCCTGCTCCAATTGC-3′
dhhc-6 Rev	5′-***GGGGACCACTTTGTACAAGAAAGCTGGG***TCTTCAGTAGTAGCCGTATCCTGGG-3′
dhhc-8 Fwd	5′-***GGGGACAAGTTTGTACAAAAAAGCAGGCT***GTGTAAACGGATATCAGCATTATTGCC-3′
dhhc-8 Rev	5′-***GGGGACCACTTTGTACAAGAAAGCTGGG***TGCGGAAGGTGCTGTAAGATAG-3′
dhhc-13 Fwd	5′-***GGGGACAAGTTTGTACAAAAAAGCAGGCT***GATGGCTGCTGATAAGAGTTTCGC-3′
dhhc-13 Rev	5′-***GGGGACCACTTTGTACAAGAAAGCTGGG***TATCTCGATGGTAGAGACATGACGAG-3′
dhhc-14 Fwd	5′-***GGGGACAAGTTTGTACAAAAAAGCAGGCT***AACCTTATTGGTGGCGTCTTGG-3′
dhhc-14 Rev	5′-***GGGGACCACTTTGTACAAGAAAGCTGGG***TCGCATAATGACTTGTAGCCATGCAG-3′
spe-10 Fwd	5′-***GGGGACAAGTTTGTACAAAAAAGCAGGCT***CGTTCAGTCAACAATTCAAGCAACC-3′
spe-10 Rev	5′-***GGGGACCACTTTGTACAAGAAAGCTGGG***TCGAACAGCACCTTCTAGTGAAGTG-3′

Additional File 15. Primers used to clone RNAi vectors. The sequences of the oligonucleotides used for cloning RNAi vectors not already available are shown. The attB sites are shown in bold italic and the annealing sequences in normal type.
